# Identifying Genetic Variants in Patients With Cefaclor‐Induced Anaphylaxis Using Human Leukocyte Antigen Typing and Whole‐Exome Sequencing

**DOI:** 10.1002/clt2.70103

**Published:** 2025-09-20

**Authors:** Sung‐Ryeol Kim, Da Eun Lee, Hyun Young Jung, In‐Wha Kim, Hye‐Ryun Kang, Kyung Hee Park, Jung‐Won Park, Jung‐Mi Oh, Jae‐Hyun Lee

**Affiliations:** ^1^ Division of Pulmonology Allergy and Critical Care Medicine Department of Internal Medicine Yongin Severance Hospital Yonsei University College of Medicine Yongin‐si South Korea; ^2^ College of Pharmacy Seoul National University Seoul South Korea; ^3^ Research Institute of Pharmaceutical Sciences Seoul National University Seoul South Korea; ^4^ Department of Internal Medicine Seoul National University College of Medicine Seoul South Korea; ^5^ Institute of Allergy Yonsei University College of Medicine Seoul South Korea; ^6^ Division of Allergy and Immunology Department of Internal Medicine Yonsei University College of Medicine Seoul South Korea; ^7^ Natural Products Research Institute Seoul National University Seoul South Korea

**Keywords:** anaphylaxis, cefaclor, genetic variation

## Abstract

**Background:**

Cefaclor is a commonly prescribed β‐lactam antibiotic and a known major cause of immediate‐type drug hypersensitivity in Korea. However, its genetic risk factors remain poorly understood. We aimed to identify genetic variants associated with cefaclor‐induced anaphylaxis and evaluate their potential clinical implications.

**Methods:**

Whole‐exome sequencing and HLA genotyping were performed in 33 patients with cefaclor‐induced anaphylaxis and 41 drug‐tolerant controls. Associations were assessed using logistic regression. Selected variants were validated in an independent Korean population. Gene set enrichment analysis (GSEA) was performed using association statistics from all variants to investigate relevant biological pathways.

**Results:**

A rare missense variant, rs765144578 in *TPSAB1* was strongly associated with anaphylaxis and remained significant in the validation control group. It was found in 90.91% of patients with hypotension, suggesting a link to reaction severity. Rs192498095 in HLA‐DRB5 showed a significant association in the discovery cohort. However, it was not detected in the replication set, likely due to its rarity and polymorphic nature. Co‐occurrence of rs765144578 in *TPSAB1* and rs192498095 in *HLA‐DRB5* markedly increased risk. GSEA revealed significant enrichment of the TNF‐α signaling via NF‐κB pathway, reflecting pathway‐level immune activation.

**Conclusion:**

Genetic variants in *TPSAB1* and *HLA‐DRB5* may contribute to the risk of cefaclor‐induced anaphylaxis, and *TPSAB1* may also be associated with severity. These findings may support the development of future screening strategies or individualized risk prediction models in β‐lactam allergy.

## 1 Introduction

Drugs are the most common triggers of anaphylaxis in adults [1], and drug‐induced anaphylaxis carries a higher risk of severe outcomes than other causes [2]. Frequent culprits include antibiotics, nonsteroidal anti‐inflammatory drugs (NSAIDs), and contrast media. Although studies have examined clinical risk factors, predicting drug‐induced anaphylaxis in individuals without a prior history of drug allergy remains challenging [3, 4, 5].

Cefaclor, a widely prescribed second‐generation cephalosporin, is one of the leading causes of drug‐induced anaphylaxis in Korea [6, 7]. Notably, cefaclor is associated with a higher incidence of anaphylaxis than other cephalosporins, making it a significant clinical concern [8, 9]. Because antibiotics are widely prescribed, overall exposure is high and may contribute to the observed frequency of hypersensitivity reactions [10] However, prescription volume alone does not fully explain why cefaclor accounts for a disproportionate share of drug‐induced anaphylaxis cases in Korea [6].

The high incidence of cefaclor‐induced hypersensitivity in Korea may reflect genetic susceptibility, including variations in HLA alleles that affect immune recognition of the drug. Identifying these genetic factors could facilitate the prediction and prevention of severe hypersensitivity reactions in at‐risk individuals. Although the intricate interactions among immune cells and signaling pathways in immediate hypersensitivity can limit the practical application of individual genetic variants, research into their genetic basis remains valuable. Such investigations provide key insights into the mechanisms of drug‐induced anaphylaxis and may support the development of personalized preventive strategies. Accordingly, we aimed to identify genetic variants associated with cefaclor‐induced anaphylaxis by performing whole‐exome sequencing (WES) and HLA genotyping.

## 2 Methods

### 2.1 Study Participants and Data Collection

We enrolled 33 adult patients (≥ 18 years old) with cefaclor‐induced anaphylaxis and 41 cefaclor‐tolerant controls (Table S1). Patients were recruited from three hospitals in Korea between 2021 and 2022, based on clinical history, oral provocation testing, specific IgE testing to cefaclor, or skin testing. Causality was assessed using the World Health Organization (WHO)‐Uppsala Monitoring Center criteria, and only cases classified as “certain” were included [11]. Anaphylaxis was diagnosed according to the World Allergy Organization (WAO) 2020 criteria [12].

Tolerant controls were recruited from the general population via hospital‐based posters and included individuals who had taken cefaclor at least twice, with intervals of more than 2 weeks, without developing significant hypersensitivity reactions. Their tolerance status was verified through a review of medical history.

The study protocol was approved by the Institutional Review Board of Severance Hospital, Yonsei University Health System (approval number: 9‐2020‐0124). Written informed consent was obtained from all participants before study enrollment.

### 2.2 Sample Preparations and WES

Genomic deoxyribonucleic acid (DNA) was extracted from 74 whole blood samples using the GeneAll Exgene Blood SV mini kit (GeneAll Biotechnology, Seoul, Korea), following the manufacturer's instructions. DNA concentration and quality were assessed using a Qubit fluorometer (Thermo Fisher Scientific, Waltham, MA, USA), and purity was verified with A260/280 ratios between 1.5 and 2.2. DNA integrity was confirmed by a DNA integrity number (DIN) > 6.

WES was performed using the NovaSeq 6000 platform (Illumina Inc., San Diego, CA, USA) with a target mean depth of 100 ×, aiming to capture variants across exons and regulatory intronic regions, including the highly polymorphic HLA region. Raw read quality was assessed using FastQC (v0.11.7) [13], and low‐quality bases (*Q* < 20) were trimmed using Trimmomatic (v0.36) [14]. Clean reads were aligned to the human reference genome GRCh37 (hg19), obtained from the UCSC Genome Browser, using Burrows‐Wheeler Aligner (BWA) v0.7.12 [15, 16, 17].

Variant calling for single nucleotide variants (SNVs) and insertions/deletions (INDELs) was conducted using the Genome Analysis Toolkit (GATK) v4.0.11.0, and functional annotation was performed with SnpEff v4.3 [18, 19].

### 2.3 Next‐Generation Sequencing (NGS)‐Based Typing of HLA Alleles

Following WES, HLA genotyping was performed to identify specific alleles in each participant. The *HLA‐A, ‐B, ‐C, ‐DPA1, ‐DPB1, ‐DQA1, ‐DQB1, ‐DRA,* and *‐DRB1* loci were typed using HISAT‐genotype v1.3.3 [20]. *HLA‐DRB3, ‐DRB4, and ‐DRB5* were genotyped using NGSgo‐AmpX v2 (GenDx, Netherlands) following the manufacturer's protocol. Sequence reads were aligned, and alleles were assigned using NGSengine software (GenDx, Netherlands), referencing the IMGT/HLA database [21]. Allele frequencies were calculated for each HLA locus in both the anaphylaxis and tolerant control groups.

### 2.4 Statistical Methods for Demographic Characteristics

Statistical analyses were conducted to compare clinical characteristics between patients with cefaclor‐induced anaphylaxis and control subjects. Continuous variables (e.g., age, BMI) were analyzed using Student's t‐test to evaluate differences between group means. Categorical variables (e.g., sex, underlying diseases) were analyzed using Fisher's exact test or the chi‐square test to assess differences in proportions between groups. Statistical significance was set at *p* < 0.05.

### 2.5 WES‐Based Association Analysis

Prior to association analysis, quality control criteria were applied to both individual‐level and variant‐level WES data. No individuals were excluded based on genotype call rate, as all participants met the predefined threshold of ≥ 90%. At the variant level, filtering was performed using bcftools (v1.13) on the VCF files [22]. Variants were retained if they had a genotype quality (GQ) score ≥ 30 and a read depth (DP) ≥ 10. The resulting data were converted into binary format using whole‐genome analysis toolset (PLINK v2.0) [23, 24]. To minimize false‐positive associations, only biallelic single nucleotide variants (SNVs) with a minor allele frequency (MAF) ≥ 0.01 and Hardy–Weinberg equilibrium (HWE) *p* value > 0.05 were included. After quality control filtering, a total of 197,422 variants across autosomal chromosomes (1–22) remained in the final dataset. To account for potential population stratification, principal component analysis (PCA) was conducted using the SNPRelate package in R (v4.3.1, R Foundation for Statistical Computing, Vienna, Austria).

Genotype–phenotype associations between 33 anaphylaxis patients and 41 tolerant controls were tested using logistic regression under an additive genetic model, implemented in PLINK v2.0. Based on principles of causal inference, none of the clinical variables were considered to temporally or causally precede genetic predisposition. Therefore, we estimated the total effect of genetic variants on the phenotype by adjusting for sex as the sole covariate, without controlling for mediators or downstream clinical traits. Variants were considered statistically significant based on a false discovery rate (FDR)‐adjusted *p* value < 0.01. Among the significant variants, candidates were prioritized as functionally relevant if predicted to be deleterious by SIFT (score < 0.05) or damaging by PolyPhen‐2 (HVAR score > 0.5) [25, 26].

To evaluate the reproducibility of the associations identified in the discovery phase, we conducted a validation analysis using an independent control group. Whole‐genome sequencing (WGS) data from 232 unrelated Korean individuals—sourced from the Korean Genome Project and provided by the Korean Genomics Center at the Ulsan National Institute of Science and Technology (UNIST), Republic of Korea—served as the independent control cohort [27]. As these WGS data were aligned to the hg38 reference genome, the top functional variants were converted to hg19 coordinates using the lift‐over tool available through the UCSC Genome Browser [17]. For the validation analysis, univariate logistic regression assuming an additive genetic model was performed in R to evaluate the association of the top functional variants. Genotype dosage differences were tested between the 33 cases from the discovery analysis and the 232 independent controls. Variants were considered replicated if they demonstrated statistically significant associations (*p* < 5 × 10^−8^) in the same direction as in the discovery analysis.

### 2.6 Pathway Enrichment Analysis

Pathway‐level enrichment was assessed using pre‐ranked gene set enrichment analysis (GSEA), implemented via the Broad Institute's GSEA software (v4.3.2) [28]. A ranked gene list was generated by ordering genes based on the lowest *p* value from the association analysis. In cases where multiple variants mapped to the same gene, only the variant with the smallest *p* value was used to represent that gene in the ranked list. Enrichment analysis was performed using the Hallmark gene sets from the Molecular Signatures Database (MSigDB v2023.2) [29]. Default parameters were applied, including 1000 permutations, a weighted enrichment statistic, and gene set size filters ranging from a minimum of 3 to a maximum of 500 genes. Nominal *p* values were used to evaluate enrichment, and gene sets with *p* < 0.05 were considered suggestively enriched.

### 2.7 Association Analysis for HLA Genotypes

For HLA association analysis, 15 of 169 typed alleles were selected based on a minimum phenotype count of five and at least a threefold difference in allele frequency between cases and tolerant controls. Associations were tested under a dominant genetic model using Fisher's exact test or the chi‐square test in R (version 4.3.1). Statistical significance was defined as *p* < 0.05.

### 2.8 Subgroup Analysis

To further explore phenotype‐specific genetic associations, two targeted subgroup analyses were carried out. The first assessed the association between the top variant in the tryptase gene, rs765144578 in *TPSAB1*, and hypotension, a severe clinical manifestation of anaphylaxis. Patients with anaphylaxis who presented with hypotension were classified into the hypotension subgroup. The second analysis evaluated the risk of anaphylaxis among individuals carrying both rs765144578 in *TPSAB1* and rs192498095 in *HLA‐DRB5*, compared to those carrying neither variant. All association analyses were performed using logistic regression models adjusted for sex.

## 3 Results

### 3.1 Clinical Characteristics of Participants

The mean age of the patients was 46.7 years, and 26 patients (78.8%) were female. Significant differences were observed between the anaphylaxis and control groups in terms of age and history of food allergy. The most common clinical manifestations among patients were cutaneous symptoms (93.3%), followed by respiratory (66.7%), cardiovascular (42.4%), and gastrointestinal (30.3%) symptoms. Serum‐specific IgE (sIgE) to cefaclor was measured in 32 patients, all of whom tested positive (> 0.35 kUA/L). One additional patient, who had positive results on both the skin test and oral provocation test, did not undergo sIgE testing, resulting in no available sIgE data for that case. Detailed clinical features of the enrolled patients and controls are summarized in Table S1.

### 3.2 Identification of Genetic Risk Variants via WES

**FIGURE 1:**
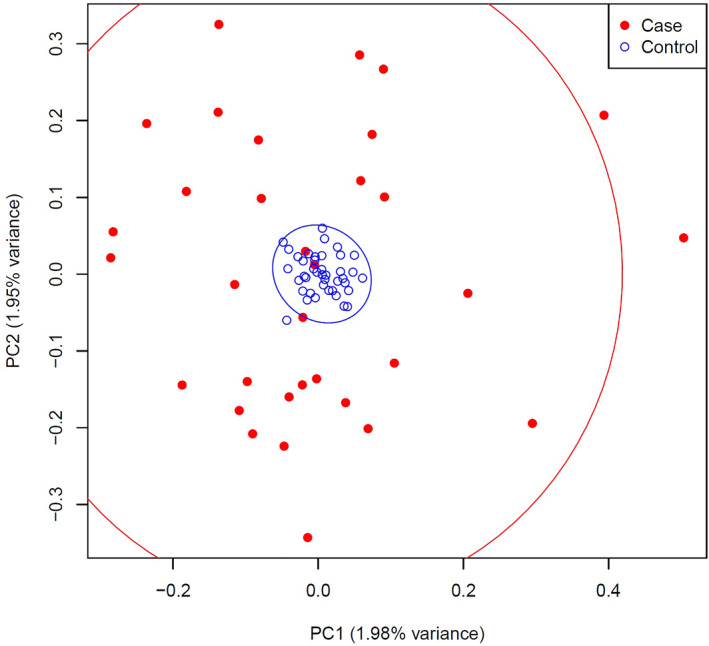
Principal component analysis of genetic variants in patients with cefaclor‐induced anaphylaxis and tolerant controls. PCA was performed using 197,422 variants derived from 33 patients with cefaclor‐induced anaphylaxis (red dots) and 41 tolerant controls (blue circles), plotted against the first two principal components (PC1 and PC2). The large dispersion and substantial overlap of confidence ellipses demonstrate limited population differentiation.

Figure 1 presents a PCA of 197,422 genetic variants in 33 patients with cefaclor‐induced anaphylaxis and 41 cefaclor‐tolerant controls. PC1 and PC2 explained 1.98% and 1.95% of the total genetic variance, suggesting minimal separation along the major axes of variation and indicating limited evidence of population stratification. Although the anaphylaxis group showed slightly greater dispersion compared to controls, the substantial overlap between groups and broad confidence ellipses indicate no clear genetic differentiation. Therefore, PCA‐derived principal components were not included as covariates in the association analysis.

**FIGURE 2:**
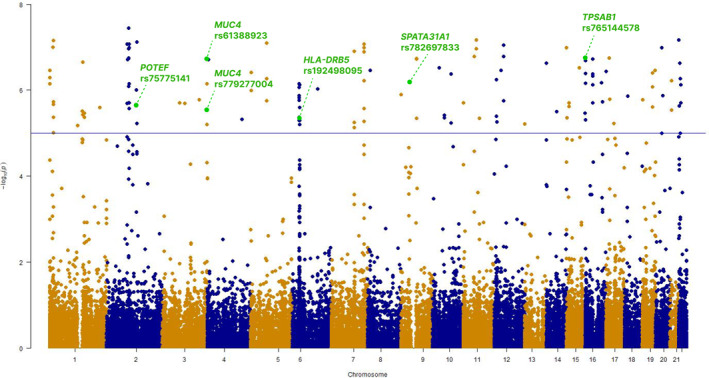
Manhattan plot of genetic association study in cefaclor‐induced anaphylaxis. Each dot represents the negative log_10_ *P* of 197,422 variants derived from a logistic regression model adjusted for sex, comparing 33 patients with cefaclor‐induced anaphylaxis and 41 tolerant controls. The blue horizontal line indicates the significance threshold, which was set at a false discovery rate (FDR)‐corrected *p* < 0.01. Green dots highlight the top six functionally annotated single nucleotide polymorphisms (SNPs), annotated with corresponding gene name and rsIDs.

During the discovery phase, genetic association analysis identified multiple clusters of variants across several genomic regions (Figure 2). Association analysis, conducted via logistic regression adjusted for sex, revealed 164 candidate variants across chromosomes 1 to 22 that met the significance threshold (FDR‐adjusted *p* < 0.01; Table S2). The Manhattan plot highlighted prominent peaks, prompting further investigation into functionally relevant variants that may influence gene expression or phenotype.

Among these 164 variants, six single nucleotide polymorphisms (SNPs) were prioritized as functionally significant based on predicted pathogenicity (SIFT score < 0.05 or PolyPhen‐2 HVAR score > 0.5) (Table 1). Notably, rs765144578 variant in the *TPSAB1* locus demonstrated the strongest association with cefaclor‐induced anaphylaxis (adjusted *p* < 0.001; risk allele frequency [RAF] 0.44 vs. 0.05; odds ratio [OR] 98.96; 95% confidence interval [CI], 17.65–555.04).

**TABLE 1:** Top six functional variants associated with cefaclor‐induced anaphylaxis identified by genetic association analysis.

Chr	Position	Variant	Gene	REF	RA	Discovery phase (*N* = 74)					Validation control (*N* = 232)		
						RAF		Number of risk allele carriers (patients/controls)	OR (95% CI)	*P*_adjusted_ ^a^	RAF	OR (95% CI)	*p* value
						Case (*N* = 33)	Tolerant (*N* = 41)						
2	130832292	rs75775141	*POTEF*	T	A	0.71	0.12	33/6	14.89 (4.86–45.65)	0.004	0.54	7.78 (3.41–17.77)	1.13 × 10^−6^
3	195507475	rs779277004	*MUC4*	G	C	0.38	0.05	25/4	45.87 (9.24–227.81)	0.004	0.24	3.35 (1.45–7.73)	4.65 × 10^−3^
3	195508010	rs61388923	*MUC4*	C	A	0.39	0.05	26/4	35.11 (9.20–133.99)	< 0.001	—	—	—
6	32489852	rs192498095	*HLA‐DRB5*	A	G	0.33	0.04	22/3	25.84 (6.44–103.74)	0.006	—	—	—
9	39890309	rs782697833	*SPATA31A1*	G	A	0.96	0.15	32/6	14.64 (5.09–42.11)	0.002	—	—	—
16	1291623	rs765144578	*TPSAB1*	C	T	0.44	0.05	29/4	98.96 (17.65–555.04)	< 0.001	0.08	38.21 (12.68–115.11)	9.51 × 10^−11^ ^b^

Abbreviations: Chr, chromosome; CI, confidence interval; OR, odds ratio; RA, risk allele; RAF, risk allele frequency; REF, reference allele.

^a^ FDR‐adjusted *p* values were derived from logistic regression analyses adjusted for sex, comparing 33 patients with cefaclor‐induced anaphylaxis and 41 tolerant controls.

^b^ Statistically significant at *p* < 5 × 10^−8^.

Other functionally relevant variants included:

- rs779277004 variant in *MUC4* (RAF 0.38 vs. 0.05; OR 45.87; 95% CI, 9.24–227.81),
- rs61388923 variant in *MUC4* (RAF 0.39 vs. 0.05; OR 35.11; 95% CI, 9.20–133.99),
- rs192498095 variant in *HLA‐DRB5* (RAF 0.33 vs. 0.04; OR 25.84; 95% CI, 6.44–103.74),
- rs75775141 variant in *POTEF* (RAF 0.71 vs. 0.12; OR 14.89; 95% CI, 4.86–45.65),
- rs782697833 variant in *SPATA31A1* (RAF 0.96 vs. 0.15; OR 14.64; 95% CI, 5.09–42.11).

Validation analysis using genotype data from 232 individuals in the general population confirmed the association of rs765144578 in *TPSAB1* with cefaclor‐induced anaphylaxis (*p* = 9.51 × 10^−11^; RAF 0.44 vs. 0.08; OR 38.20; 95% CI, 12.68–115.11). While rs75775141 in *POTEF* and rs779277004 in *MUC4* showed associations in the same direction, they did not reach statistical significance in the validation dataset. The remaining three variants could not be evaluated due to their low allele frequencies.

### 3.3 Pathway Enrichment Analysis via GSEA

GSEA revealed signaling pathways potentially contributing to the pathogenesis of cefaclor‐induced anaphylaxis (Table 2). Among the analyzed gene sets, TNF‐α signaling via the NF‐κB pathway was significantly enriched (188 genes; normalized enrichment score [NES] = 1.20; nominal *p* = 0.017). Other pathways, including IL‐6 JAK STAT3 signaling (NES = 1.14; *p* = 0.147) and oxidative phosphorylation (NES = 1.10; *p* = 0.143), also exhibited positive enrichment trends but without statistical significance. Detailed gene lists and enrichment scores for the TNF‐α signaling via NF‐κB pathway are provided in Table S3.

**TABLE 2:** Gene set enrichment analysis of variants associated with cefaclor‐induced anaphylaxis.

Identifier	Gene set pathway annotation	Gene set size^a^	Normalized enrichment score	*p* value
M5890	TNF‐α signaling via NF‐κB	188	1.20	0.017^b^
M5897	IL‐6 JAK STAT3 signaling	78	1.14	0.147
M5928	MYC targets V2	54	1.14	0.198
M5936	Oxidative phosphorylation	174	1.10	0.143
M5926	MYC targets V1	180	1.08	0.214
M5932	Inflammatory response	191	1.07	0.238

*Note:* Gene sets were tested for enrichment using pre‐ranked gene set enrichment analysis (GSEA) with Hallmark gene sets from Molecular Signatures Database (MSigDB).

^a^ Gene set size refers to the number of genes in the pathway that were present in the pre‐ranked input list.

^b^ Gene sets with nominal *p* < 0.05 were considered suggestively enriched.

### 3.4 HLA Alleles Associated With Anaphylaxis

NGS‐based HLA typing revealed specific alleles associated with cefaclor‐induced anaphylaxis (Table 3). The overall distribution of HLA alleles observed in the study population is shown in Figure S1. Among these, the HLA‐B*07:02 was significantly associated with an increased risk of anaphylaxis, being present in 5 out of 33 patients but absent in all 41 tolerant controls *(p* = 0.015; OR = 7.15; 95% CI, 1.22–353.85). In contrast, HLA‐C*01:02 appeared to have a protective effect, being present in 4 anaphylaxis cases and 14 tolerant controls (*p* = 0.033; OR = 0.27; 95% CI, 0.06–0.99). Although not statistically significant, several HLA class II alleles including DQB1*03:03, DQB1*05:01, and DRB5*01:01 showed notable differences in frequency between cases and controls.

**TABLE 3:** HLA alleles associated with cefaclor‐induced anaphylaxis.

HLA types	Dominant genotype frequency		OR^a^ (95% CI)	*p* value
	Cases	Controls		
B * 07:02	0.15	0	7.15 (1.22–353.85)	0.015^b^
B * 46:01	0.06	0.15	0.38 (0.04–2.33)	0.286
B * 54:01	0.03	0.12	0.23 (0–2.20)	0.216
B * 58:01	0.06	0.15	0.38 (0.04–2.33)	0.286
C * 01:02	0.12	0.34	0.27 (0.06–0.99)	0.033^b^
C * 03:02	0.06	0.15	0.38 (0.04–2.33)	0.286
DQA1 * 01:01	0.18	0.05	4.25 (0.69–46.14)	0.128
DQA1 * 01:03	0.09	0.24	0.31 (0.05–1.38)	0.125
DQB1 * 03:03	0.09	0.27	0.28 (0.05–1.19)	0.074
DQB1 * 05:01	0.21	0.05	5.14 (0.89–54.43)	0.069
DQB1 * 06:02	0.09	0.24	0.31 (0.05–1.38)	0.125
DRB1 * 01:01	0.18	0.05	4.25 (0.69–46.14)	0.128
DRB1 * 08:03	0.06	0.17	0.32 (0.03–1.84)	0.283
DRB1 * 15:01	0.09	0.24	0.31 (0.05–1.38)	0.125
DRB5 * 01:01	0.09	0.27	0.28 (0.05–1.19)	0.074

Abbreviations: CI, confidence interval; HLA, human leukocyte antigen; OR, odds ratio.

^a^ Odds ratios and 95% confidence intervals were calculated under a dominant model using Fisher's exact test, with Haldane's correction applied where necessary.

^b^ Statistically significant at *p* < 0.05.

### 3.5 Evaluation of Top Variants in Clinical Subgroups

Subgroup analysis revealed a strong association between the rs765144578 variant in *TPSAB1* and hypotension as a manifestation of anaphylaxis (Table 4). Among the 11 patients who experienced hypotension, 10 (90.91%) carried the variant, compared with 4 of 41 tolerant controls (9.76%), yielding an OR of 130.71 (*p* = 1.13 × 10^−4^). To further investigate this association, the frequency of the rs765144578 variant was compared between patients with hypotension and individuals from the general Korean population, in which 16.0% (37/232) carried the variant. This comparison yielded an OR of 52.70 (*p* = 1.94 × 10^−4^), further supporting its potential involvement in severe presentations of immediate hypersensitivity reactions.

**TABLE 4:** Association between the rs765144578 variant in TPSAB1 and hypotension in patients with cefaclor‐induced anaphylaxis.

Clinical symptoms	Cases with symptoms	Tolerant controls			Validation controls		
	GF	GF	OR (95% CI)	*P*_adjusted_ ^a^	GF	OR (95% CI)	*p* value
Hypotension	10/11 (90.91%)	4/41 (9.8%)	130.71 (11.02–1550.47)	1.13 × 10^−4^	37/232 (16.0%)	52.70 (6.55–424.16)	1.94 × 10^−4^

Abbreviations: CI, confidence interval; GF, genotype frequency (Number of risk allele carriers); OR, odds ratio.

^a^ *p* values from the sex‐adjusted additive logistic regression model.

Furthermore, the co‐occurrence of rs765144578 variant in *TPSAB1* and rs192498095 variant in *HLA‐DRB5* demonstrated a markedly stronger association with anaphylaxis than either variant alone. Individuals carrying both variants were significantly more frequent in the anaphylaxis group than in tolerant controls (57.6% vs. 2.4%), with an OR of 657.38 (95% CI, 38.87–11,119; *p* < 0.001), suggesting a potential synergistic effect between the two loci.

## 4 Discussion

Previous genetic studies on drug hypersensitivity have predominantly focused on delayed‐type reactions. However, an increasing number of genetic factors have also been associated with immediate hypersensitivity. For example, polymorphisms in cytokine‐related genes—such as TNF‐α, IL‐4, IL‐13, IL‐10, and IL‐18—as well as in the FcεRI receptor on mast cells, have been linked to immediate hypersensitivity reactions to β‐lactam antibiotics [30, 31, 32, 33, 34]. In addition, genome‐wide association studies (GWAS) have reported associations with various HLA genes, including HLA‐B, HLA‐DRA, HLA‐DRB1*04:03, HLA‐DRB1*10:01, HLA‐DRB1*14:54, and HLA‐B*55:01 [35, 36, 37, 38]. Although these genetic markers have not yet proven to be practically useful for predicting drug‐induced hypersensitivity, they have provided valuable insights into the immunogenetic mechanisms underlying anaphylaxis. Building on this background, we aimed to elucidate the genetic mechanisms specific to cefaclor‐induced anaphylaxis and explore their potential clinical relevance.

This study explores genetic predispositions to cefaclor‐induced anaphylaxis at both the exome‐wide and HLA region levels, focusing specifically on patients with confirmed anaphylaxis rather than general hypersensitivity. Through genetic association study, we identified 164 variants meeting the significance threshold (FDR‐adjusted *p* < 0.01). Among these, six variants were prioritized based on predicted pathogenicity using PolyPhen‐2 and SIFT algorithms. The genes harboring these variants included *POTEF, MUC4, SPATA31A1, HLA‐DRB5*, and *TPSAB1*.

Three of these genes—*POTEF, MUC4*, and *SPATA31A1*—showed higher variant frequencies in patients compared to tolerant controls. However, based on their known biological roles—*POTEF* in retinal homeostasis [39], *MUC4* in mucin production [40], and *SPATA31A1* in cell differentiation and spermatogenesis [41]—it is unlikely that these genes are directly involved in the pathogenesis of cefaclor‐induced anaphylaxis.

In contrast, the *HLA‐DRB5* and the *TPSAB1* are more plausibly implicated in drug induced anaphylaxis. *HLA‐DRB5*, a class II HLA gene, has previously been associated with IgE sensitization [42], while *TPSAB1* encodes α‐ and β‐tryptases. β‐tryptase, the most abundant mediator stored in mast cell granules, plays a central role in allergic responses by amplifying mast cell degranulation and enhancing inflammatory signaling [43].

To validate the associations identified in the initial case–control analysis, we conducted a secondary analysis using whole‐genome sequencing data from healthy unrelated Korean individuals. The rs765144578 variant in *TPSAB1* remained significantly associated with cefaclor‐induced anaphylaxis, reaching genome‐wide significance (*p* < 1 × 10^−8^). In contrast, the variants in *HLA‐DRB5, MUC4,* and *SPATA31A1* were not detected in the validation dataset, possibly due to low allele frequencies, differences in sequencing depth, or quality control filtering. Taken together, *TPSAB1* and *HLA‐DRB5* emerge as meaningful genetic contributors to cefaclor‐induced anaphylaxis based on both biological plausibility and case–control association signals. However, the lack of replication for *HLA‐DRB5* in the validation cohort warrants cautious interpretation of its role. The rarity and polymorphic nature of the variant, along with differences in analytic conditions between the discovery and validation datasets, may have contributed to the absence of rs192498095 in *HLA‐DRB5* in the replication analysis.

HLA association analysis identified HLA‐B*07:02 as a potential risk allele and HLA‐C*01:02 as a possible protective allele in cefaclor‐induced anaphylaxis. Although a direct mechanistic link between HLA class I alleles and allergen sensitization has not been well established, several studies have reported associations between HLA class I alleles and drug‐induced immediate hypersensitivity reactions. For instance, beta‐lactam‐induced hypersensitivity has been associated with HLA‐B*48:01, HLA‐C*04:06, and HLA‐C*08:01 [44]. Similarly, HLA‐B*46:01 has been implicated in asparaginase‐induced hypersensitivity, while HLA‐B*38:02 and HLA‐B*58:01 have been associated with contrast media‐induced hypersensitivity [45, 46]. These precedents support the plausibility of associations between HLA‐B*07:02, HLA‐C*01:02, and cefaclor‐induced immediate hypersensitivity.

A recent study identified HLA‐DRB1*04:03 and HLA‐DRB1*14:54 as risk factors for cefaclor‐induced immediate hypersensitivity [36]. In our cohort, HLA‐DRB1*04:03 was observed in three patients but not in any tolerant controls, while HLA‐DRB1*14:54 was found in one patient and three tolerant controls, with no statistically significant difference. Given the small number of individuals carrying these alleles, a reanalysis in a larger cohort is warranted to clarify their potential roles. Additionally, a separate study recently reported HLA‐DRB3*02:02 as a risk factor for penicillin hypersensitivity [47]. In our cohort, this allele was present in nine patients and eleven tolerant controls, again showing no significant difference. It is noteworthy that the previous association was observed in the context of delayed hypersensitivity, whereas our study focused on immediate hypersensitivity. This difference in immunopathological context may partly explain the lack of concordant findings.

We identified an association between the rs192498095 variant in *HLA‐DRB5* and cefaclor‐induced anaphylaxis based on WES data. However, HLA genotyping did not reveal a specific *HLA‐DRB5* allele significantly associated with the phenotype. Among the alleles examined, *HLA‐DRB5∗01:01* showed the greatest frequency difference—being more common in controls than in patients—but this difference was not statistically significant (OR = 0.28, *p* = 0.074). Linkage disequilibrium analysis also showed low correlation (*r* ^2^ = 0.13) between *HLA‐DRB5∗01:01* and the reference allele A of rs192498095, suggesting a weak relationship between the SNP and this classical allele. Since classical HLA alleles are typically determined by combinations of multiple variants, the observed discrepancy suggests that rs192498095 may reflect a regulatory or non‐classical signal not directly tied to a specific *HLA‐DRB5* allele.

Genetic variability in *TPSAB1* has previously been associated with the severity of hypersensitivity reactions [48, 49]. In our subgroup analysis, the rs765144578 variant was identified in 90.91% of patients with hypotension yielding an OR of 130.71 (Table 4), which exceeded the overall OR of 98.96 observed in the total cohort. These findings suggest a strong association between this variant and the severity of cefaclor‐induced immediate hypersensitivity reactions. Given the known role of *TPSAB1* in mast cell activation and tryptase production, it is possible that this rare missense variant may potentially affect tryptase protein stability or expression, thereby enhancing mast cell degranulation and amplifying allergic inflammation [50]. The variant demonstrated consistent associations across both the discovery and validation cohorts. However, further prospective and functional studies are warranted to clarify its mechanistic role and assess its clinical relevance.

Immediate drug hypersensitivity is known to be influenced by various genetic factors [51], and the presence of multiple susceptibility variants may act synergistically to increase the likelihood of reaction [52]. In our subgroup analysis, the co‐occurrence of the *TPSAB1* variant rs765144578 and the *HLA‐DRB5* variant rs192498095 was strongly associated with cefaclor‐induced anaphylaxis (Table 5; OR = 657.38), suggesting a combined contribution of pathways involving IgE sensitization and tryptase‐mediated mast cell activation to the pathogenesis of this condition. In addition, TNF‐α signaling may also play a critical role in the development of cefaclor‐induced anaphylaxis. Both GSEA and genetic association analysis revealed significant enrichment of the TNF‐α signaling via the NF‐κB pathway in affected individuals. The top six functional variants identified by GWAS were not mapped to any of the significantly enriched pathways, including the TNF‐α signaling set. This suggests that these variants may exert their pathogenic effects through independent or complementary mechanisms. Although no single GWAS‐identified variant directly overlapped with the TNF‐α pathway, the GSEA findings point to an aggregated immune activation signal at the pathway level. This finding aligns with previous reports linking TNF‐α gene variants to immediate hypersensitivity reactions to β‐lactam antibiotics [30]. TNF is produced during the early stages of allergen sensitization and is essential for the production of antigen‐specific IgE [53]. It also plays a key role in mast cell activation [54], wherein NF‐κB serves as a central transcription factor mediating TNF‐α signaling [55]. The observed enrichment of this pathway raises the possibility that polymorphisms in the TNF promoter region may modulate TNF‐α expression, thereby contributing to amplified allergic inflammation.

**TABLE 5:** Synergistic effect of rs192498095 and rs765144578 variants on the risk of cefaclor‐induced anaphylaxis.

Variants	Cefaclor anaphylaxis (*N* = 33)	Tolerant control (*N* = 41)	OR (95% CI)	*P*_adjusted_ ^a^
rs192498095(+)/rs765144578(+)	19 (57.6%)	1 (2.4%)	657.38 (38.87–11119)	6.91 × 10^−6^
rs192498095(−)/rs765144578(−)	1 (3.0%)	35 (85.4%)		

Abbreviations: CI, confidence interval; OR, odds ratio.

^a^ *p*‐values from the sex‐adjusted additive logistic regression model.

The present study had several limitations. First, completely ruling out sequencing or variant calling errors was difficult due to the highly polymorphic nature of the HLA region. To mitigate this, we employed a target depth of 100 × and implemented stringent quality control procedures. Second, the rs765144578 variant in *TPSAB1* may be indirectly linked to hereditary alpha tryptasemia (HαT), potentially contributing to increased severity of hypersensitivity. However, we were unable to assess serum tryptase levels or determine *TPSAB1* copy number, which limits our ability to definitively exclude this possibility. Nevertheless, none of the patients had a clinical history suggestive of HαT, and the involvement of this single nucleotide variant in HαT pathogenesis appears unlikely. Lastly, the relatively small sample size may limit the generalizability and statistical power of some findings. Despite these limitations, the associations identified—particularly those involving *TPSAB1*—remain compelling and biologically plausible. These findings not only enhance our understanding of the genetic basis of cefaclor‐induced anaphylaxis but may also inform future clinical applications. Although routine pre‐prescription screening is not currently feasible, variants such as rs765144578 could help guide risk stratification in individuals requiring re‐administration of β‐lactam antibiotics. Furthermore, the markedly increased risk observed in individuals harboring both TPSAB1 and HLA‐DRB5 variants highlights the potential utility of polygenic risk models. Such models may offer improved predictive accuracy over single‐marker approaches and warrant validation in larger, multi‐center cohorts.

### 4.1 Conclusion

Cefaclor‐induced anaphylaxis appears to involve multiple genetic factors, with rs765144578 in *TPSAB1* showing consistent associations with both risk and severity. Although rs192498095 in HLA‐DRB5 showed a significant association in the patient cohort, it was not detected in the validation dataset, possibly due to its rarity. In addition, The HLA‐B*07:02 demonstrated a significant association, though further validation is needed. These findings provide new insight into the genetic architecture of immediate hypersensitivity to cefaclor. The strong association of TPSAB1, and its potential interaction with HLA‐DRB5, may support the future development of predictive strategies for identifying individuals at increased risk. Further validation and functional studies are warranted to explore the clinical utility and mechanistic basis of these associations.

## Author Contributions

Conceptualization: Jae‐Hyun LEE, Jung‐Mi Oh, Sung‐Ryeol Kim. Methodology: Sung‐Ryeol KIM, Jae‐Hyun LEE, Jung‐Mi Oh, Jung‐Won Park. Software: Da Eun Lee. Data curations: Sung‐Ryeol Kim, Hye‐Ryun Kang, Kyung Hee Park. Investigation: Da Eun Lee, Hyun Young Jung, In‐Wha Kim. Validation: Da Eun Lee, Hyun Young Jung, In‐Wha Kim, Kyung Hee Park. Formal analysis: Da Eun Lee, Hyun Young Jung. Supervision: Jae‐Hyun LEE, Jung‐Mi Oh. Funding acquisition: Jae‐Hyun LEE, Jung‐Mi Oh. Visualization: Da Eun Lee, Hyun Young Jung. Project administration: Jae‐Hyun LEE, Jung‐Mi Oh. Resources: Jae‐Hyun LEE, Hye‐Ryun Kang, Jung‐Mi Oh, Jung‐Won Park. Writing – original draft: Sung‐Ryeol KIM, Da Eun Lee. Writing – review and editing: Jae‐Hyun LEE, Jung‐Mi Oh.

## Ethics Statement

The present study was approved by the Institutional Review Board of Severance Hospital, Yonsei University Health System (approval number: 9‐2020‐0124). Written informed consent was obtained from all participants prior to their participation in the study.

## Conflicts of Interest

The authors declare no conflicts of interest.

## Supporting information


**Figure S1:** Distribution of HLA Class I and Class II genotypes in 33 patients with cefaclor‐induced anaphylaxis.

**Figure S2:** Distribution of HLA Class I and Class II genotypes in 41 tolerant controls.

**Table S1:** Demographic and baseline clinical characteristics of patients with cefaclor‐induced anaphylaxis and tolerant controls.

**Table S2:** One hundred sixty‐four candidate variants associated with cefaclor‐induced anaphylaxis identified by exome‐wide association study.

**Table S3:** Detailed gene lists and enrichment scores for the TNF‐α signaling via NF‐κB (M5890) pathway.

## Data Availability

The genome metadata supporting the findings of this study have been deposited in the European Genome‐phenome Archive (EGA) under the accession ID EGAD50000001660 (https://ega‐archive.org/datasets/EGAD50000001660). Access to these data will be granted upon reasonable request and following data access committee approval, in accordance with ethical and privacy considerations. In addition, the analysis code and command‐line workflows used in this study are openly available on GitHub at https://github.com/DAEUN‐LEE94/cefaclor‐anaphylaxis to support reproducibility and transparency.

## References

[1] M. S. Yang , S. H. Lee , T. W. Kim , et al., “Epidemiologic and Clinical Features of Anaphylaxis in Korea,” Annals of Allergy, Asthma, & Immunology 100, no. 1 (2008): 31–36, 10.1016/s1081-1206(10)60401-2. 18254479

[2] P. J. Turner , E. Jerschow , T. Umasunthar , R. Lin , D. E. Campbell , and R. J. Boyle , “Fatal Anaphylaxis: Mortality Rate and Risk Factors,” Journal of Allergy and Clinical Immunology: In Practice 5, no. 5 (2017): 1169–1178, 10.1016/j.jaip.2017.06.031. 28888247 PMC5589409

[3] A. Banerji , R. Solensky , E. J. Phillips , and D. A. Khan , “Drug Allergy Practice Parameter Updates to Incorporate Into Your Clinical Practice,” Journal of Allergy and Clinical Immunology: In Practice 11, no. 2 (2023): 356–368, 10.1016/j.jaip.2022.12.002. 36563781

[4] D. Dubrall , N. L. Branding , C. M. Mathey , et al., “Non‐Genetic Factors Associated With ACE‐Inhibitor and Angiotensin Receptor Blocker‐Induced Angioedema,” Clinical and Translational Allergy 15, no. 5 (2025): e70058, 10.1002/clt2.70058. 40338121 PMC12058302

[5] B. Y. Thong and T. C. Tan , “Epidemiology and Risk Factors for Drug Allergy,” British Journal of Clinical Pharmacology 71, no. 5 (2011): 684–700, 10.1111/j.1365-2125.2010.03774.x. 21480948 PMC3093074

[6] K. Jeong , Y. M. Ye , S. H. Kim , et al., “A Multicenter Anaphylaxis Registry in Korea: Clinical Characteristics and Acute Treatment Details From Infants to Older Adults,” World Allergy Organization Journal 13, no. 8 (2020): 100449, 10.1016/j.waojou.2020.100449. 32817782 PMC7426446

[7] H. I. Rhyou , S. R. Kim , J. W. Jung , et al., “Clinical Characteristics and Risk Factors for Escalation to Anaphylaxis From Non‐Severe Drug Hypersensitivity Reaction,” Clinical and Translational Allergy 15, no. 4 (2025): e70047, 10.1002/clt2.70047. 40263639 PMC12014396

[8] H. I. Rhyou , Y. H. Nam , S. C. Kim , et al., “Cefaclor‐Induced Hypersensitivity: Differences in the Incidence of Anaphylaxis Relative to Other 2nd and 3rd Generation Cephalosporins,” PLoS One 16, no. 7 (2021): e0254898, 10.1371/journal.pone.0254898. 34293048 PMC8297852

[9] H. S. Yoo , S. H. Kim , H. S. Kwon , et al., “Immunologic Evaluation of Immediate Hypersensitivity to Cefaclor,” Yonsei Medical Journal 55, no. 6 (2014): 1473–1483, 10.3349/ymj.2014.55.6.1473. 25323882 PMC4205685

[10] A. L. Redel , W. Feleszko , A. Arcolaci , et al., “A Survey Study on Antibiotic Prescription Practices for Acute Asthma Exacerbations: An European Academy of Allergy and Clinical Immunology Task Force Report,” Clinical and Translational Allergy 14, no. 3 (2024): e12345, 10.1002/clt2.12345. 38497844 PMC10946284

[11] World Health Organization Uppsala Monitoring Centre , The Use of the WHO‐UMC System for Standardised Case Causality Assessment (Uppsala Monit Cent, 2005), accessed July 2025, http://www.who‐umc.org/Graphics/24734.pdf.

[12] V. Cardona , I. J. Ansotegui , M. Ebisawa , et al., “World Allergy Organization Anaphylaxis Guidance 2020,” World Allergy Organization Journal 13, no. 10 (2020): 100472, 10.1016/j.waojou.2020.100472. 33204386 PMC7607509

[13] F. Simon Andrews , A. Segonds‐Pichon , L. Biggins , C. Krueger , and S. Wingett , Fastqc: A Quality Control Tool for High Throughput Sequence Data, 2010, https://www.bioinformatics.babraham.ac.uk/projects/fastqc/.

[14] A. Kechin , U. Boyarskikh , A. Kel , and M. Filipenko , “Cutprimers: A New Tool for Accurate Cutting of Primers From Reads of Targeted Next Generation Sequencing,” Journal of Computational Biology 24, no. 11 (2017): 1138–1143, 10.1089/cmb.2017.0096. 28715235

[15] Genome Reference Consortium , Human Genome Assembly GRCh37 (Hg19) NCBI Assembly, https://www.ncbi.nlm.nih.gov/assembly/GCF_000001405.13/.

[16] H. Li and R. Durbin , “Fast and Accurate Short Read Alignment With Burrows‐Wheeler Transform,” Bioinformatics 25, no. 14 (2009): 1754–1760, 10.1093/bioinformatics/btp324. 19451168 PMC2705234

[17] W. J. Kent , C. W. Sugnet , T. S. Furey , et al., “The Human Genome Browser at UCSC,” Genome Research 12, no. 6 (2002): 996–1006, 10.1101/gr.229102. 12045153 PMC186604

[18] A. McKenna , M. Hanna , E. Banks , et al., “The Genome Analysis Toolkit: A Mapreduce Framework for Analyzing Next‐Generation DNA Sequencing Data,” Genome Research 20, no. 9 (2010): 1297–1303, 10.1101/gr.107524.110. 20644199 PMC2928508

[19] P. Cingolani , A. Platts , L. Wang le , et al., “A Program for Annotating and Predicting the Effects of Single Nucleotide Polymorphisms, Snpeff: Snps in the Genome of Drosophila Melanogaster Strain w1118; iso‐2; iso‐3,” Fly 6, no. 2 (2012): 80–92, 10.4161/fly.19695. 22728672 PMC3679285

[20] Daehwan Kim Lab , HISAT Genotype v1.3.3, 2020, https://daehwankimlab.github.io/hisat‐genotype/about/.

[21] J. Robinson , D. J. Barker , and S. G. E. Marsh , IPD‐IMGT/HLA Database (European Bioinformatics Institute/Anthony Nolan Research Institute), accessed July, 2025, https://www.ebi.ac.uk/ipd/imgt/hla/.

[22] P. Danecek , J. K. Bonfield , J. Liddle , et al., “Twelve Years of Samtools and Bcftools,” GigaScience 10, no. 2 (2021): giab008, 10.1093/gigascience/giab008. 33590861 PMC7931819

[23] C. Chang , PLINK 2.00 Alpha (2022), https://www.cog‐genomics.org/plink/2.0/.

[24] S. Purcell , B. Neale , K. Todd‐Brown , et al., “PLINK: A Tool Set for Whole‐Genome Association and Population‐Based Linkage Analyses,” American Journal of Human Genetics 81, no. 3 (2007): 559–575, 10.1086/519795. 17701901 PMC1950838

[25] P. C. Ng and S. Henikoff , “SIFT: Predicting Amino Acid Changes That Affect Protein Function,” Nucleic Acids Research 31, no. 13 (2003): 3812–3814, 10.1093/nar/gkg509. 12824425 PMC168916

[26] I. A. Adzhubei , S. Schmidt , L. Peshkin , et al., “A Method and Server for Predicting Damaging Missense Mutations,” Nature Methods 7, no. 4 (2010): 248–249, 10.1038/nmeth0410-248. 20354512 PMC2855889

[27] S. Jeon , Y. Bhak , Y. Choi , et al., “Korean Genome Project: 1094 Korean Personal Genomes With Clinical Information,” Science Advances 6, no. 22 (2020): eaaz7835, 10.1126/sciadv.aaz7835. 32766443 PMC7385432

[28] A. Subramanian , P. Tamayo , V. K. Mootha , et al., “Gene Set Enrichment Analysis: A Knowledge‐Based Approach for Interpreting Genome‐Wide Expression Profiles,” Proceedings of the National Academy of Sciences of the USA 102, no. 43 (2005): 15545–15550, 10.1073/pnas.0506580102. 16199517 PMC1239896

[29] A. Liberzon , C. Birger , H. Thorvaldsdottir , M. Ghandi , J. P. Mesirov , and P. Tamayo , “The Molecular Signatures Database (Msigdb) Hallmark Gene Set Collection,” Cell Systems 1, no. 6 (2015): 417–425, 10.1016/j.cels.2015.12.004. 26771021 PMC4707969

[30] R. M. Gueant‐Rodriguez , J. L. Gueant , M. Viola , D. Tramoy , F. Gaeta , and A. Romano , “Association of Tumor Necrosis Factor‐Alpha—308G>A Polymorphism With IgE‐Mediated Allergy to Betalactams in an Italian Population,” Pharmacogenomics Journal 8, no. 2 (2008): 162–168, 10.1038/sj.tpj.6500456. 17471286

[31] R. M. Gueant‐Rodriguez , A. Romano , M. Beri‐Dexheimer , M. Viola , F. Gaeta , and J. L. Gueant , “Gene‐Gene Interactions of IL13 and IL4RA Variants in Immediate Allergic Reactions to Betalactam Antibiotics,” Pharmacogenetics and Genomics 16, no. 10 (2006): 713–719, 10.1097/01.fpc.0000230409.00276.44. 17001290

[32] L. Guglielmi , C. Fontaine , C. Gougat , et al., “IL‐10 Promoter and IL4‐Rα Gene Snps Are Associated With Immediate Β‐Lactam Allergy in Atopic Women,” Allergy 61, no. 8 (2006): 921–927, 10.1111/j.1398-9995.2006.01067.x. 16867043

[33] L. Ming , Q. Wen , H. Qiao , and Z. Dong , “Interleukin‐18 and IL18− 607A/C And− 137G/C Gene Polymorphisms in Patients With Penicillin Allergy,” Journal of International Medical Research 39, no. 2 (2011): 388–398, 10.1177/147323001103900206. 21672342

[34] Y. H. Nam , J. E. Kim , S. H. Kim , et al., “Identifying Genetic Susceptibility to Sensitization to Cephalosporins in Health Care Workers,” Journal of Korean Medical Science 27, no. 11 (2012): 1292–1299, 10.3346/jkms.2012.27.11.1292. 23166408 PMC3492661

[35] J. L. Gueant , A. Romano , J. A. Cornejo‐Garcia , et al., “HLA‐DRA Variants Predict Penicillin Allergy in Genome‐Wide Fine‐Mapping Genotyping,” Journal of Allergy and Clinical Immunology 135, no. 1 (2015): 253–259, 10.1016/j.jaci.2014.07.047. 25224099

[36] S.‐Y. Park , S. Y. Park , S. Seo , et al., “HLA‐DRB1 Is Associated With Cefaclor‐Induced Immediate Hypersensitivity,” World Allergy Organ J 17, no. 5 (2024): 100901, 10.1016/j.waojou.2024.100901. 38638799 PMC11021981

[37] P. Nicoletti , D. F. Carr , S. Barrett , et al., “Beta‐Lactam‐Induced Immediate Hypersensitivity Reactions: A Genome‐Wide Association Study of a Deeply Phenotyped Cohort,” Journal of Allergy and Clinical Immunology 147, no. 5 (2021): 1830–1837, 10.1016/j.jaci.2020.10.004. 33058932 PMC8100096

[38] K. Krebs , J. Bovijn , N. Zheng , et al., “Genome‐Wide Study Identifies Association Between HLA‐B( *)55:01 and Self‐Reported Penicillin Allergy,” American Journal of Human Genetics 107, no. 4 (2020): 612–621, 10.1016/j.ajhg.2020.08.008. 32888428 PMC7536643

[39] (NCBI) NCfBI , Gene: POTEF POTE Ankyrin Domain Family Member F (2024), https://www.ncbi.nlm.nih.gov/gene/728378.

[40] (NCBI) NCfBI , Gene: MUC4 Mucin 4, Cell Surface Associated (2024), https://www.ncbi.nlm.nih.gov/gene/4585.

[41] (NCBI) NCfBI , Gene: SPATA31A1 Spermatogenesis Associated 31A1 (2024), https://www.ncbi.nlm.nih.gov/gene/647060.

[42] A. Oussalah , C. Mayorga , M. Blanca , et al., “Genetic Variants Associated With Drugs‐Induced Immediate Hypersensitivity Reactions: A PRISMA‐Compliant Systematic Review,” Allergy 71, no. 4 (2016): 443–462, 10.1111/all.12821. 26678823

[43] V. Payne and P. C. Kam , “Mast Cell Tryptase: A Review of Its Physiology and Clinical Significance,” Anaesthesia 59, no. 7 (2004): 695–703, 10.1111/j.1365-2044.2004.03757.x. 15200544

[44] P. Singvijarn , W. Manuyakorn , S. Mahasirimongkol , et al., “Association of HLA Genotypes With Beta‐Lactam Antibiotic Hypersensitivity in Children,” Asian Pacific Journal of Allergy & Immunology 39, no. 3 (2021): 197–205, 10.12932/AP-271118-0449. 31012593

[45] G. T. Chua , J. S. Rosa Duque , D. K. L. Cheuk , et al., “HLA Alleles Associated With Asparaginase Hypersensitivity in Chinese Children,” Journal of Hematology & Oncology 14, no. 1 (2021): 182, 10.1186/s13045-021-01201-3. 34717720 PMC8557538

[46] E. Y. Kim , S. J. Choi , J. L. Ghim , et al., “Associations Between HLA‐A, ‐B, and ‐C Alleles and Iodinated Contrast Media‐Induced Hypersensitivity in Koreans,” Translational and Clinical Pharmacology 29, no. 2 (2021): 107–116, 10.12793/tcp.2021.29.e10. 34235123 PMC8255545

[47] A. Romano , A. Oussalah , C. Chery , et al., “Next‐Generation Sequencing and Genotype Association Studies Reveal the Association of HLA‐DRB3*02:02 With Delayed Hypersensitivity to Penicillins,” Allergy 77, no. 6 (2022): 1827–1834, 10.1111/all.15147. 34687232

[48] J. J. Lyons , X. Yu , J. D. Hughes , et al., “Elevated Basal Serum Tryptase Identifies a Multisystem Disorder Associated With Increased TPSAB1 Copy Number,” Nature Genetics 48, no. 12 (2016): 1564–1569, 10.1038/ng.3696. 27749843 PMC5397297

[49] J. J. Lyons , J. Chovanec , M. P. O'Connell , et al., “Heritable Risk for Severe Anaphylaxis Associated With Increased α‐Tryptase–Encoding Germline Copy Number at TPSAB1,” Journal of Allergy and Clinical Immunology 147, no. 2 (2021): 622–632, 10.1016/j.jaci.2020.06.035. 32717252

[50] W. Kong , Y. Dong , S. Yi , W. Mo , and H. Yang , “High‐Risks Drug Adverse Events Associated With Cetirizine and Loratadine for the Treatment of Allergic Diseases: A Retrospective Pharmacovigilance Study Based on the FDA Adverse Event Reporting System Database,” Clinical and Translational Allergy 14, no. 9 (2024): e12392, 10.1002/clt2.12392. 39257032 PMC11387460

[51] C. B. Chen , R. Abe , R. Y. Pan , et al., “An Updated Review of the Molecular Mechanisms in Drug Hypersensitivity,” Journal of Immunology Research 2018 (2018): 6431694–6431722, 10.1155/2018/6431694. 29651444 PMC5830968

[52] M. Y. Kim , J. Yun , D. Y. Kang , et al., “HLA‐A*24:02 Increase the Risk of Allopurinol‐Induced Drug Reaction With Eosinophilia and Systemic Symptoms in HLA‐B*58:01 Carriers in a Korean Population; a Multicenter Cross‐Sectional Case‐Control Study,” Clinical and Translational Allergy 12, no. 9 (2022): e12193, 10.1002/clt2.12193. 36176736 PMC9478421

[53] S. Ahmad , N. A. Azid , J. C. Boer , et al., “The Key Role of TNF‐TNFR2 Interactions in the Modulation of Allergic Inflammation: A Review,” Frontiers in Immunology 9 (2018): 2572, 10.3389/fimmu.2018.02572. 30473698 PMC6238659

[54] E. Brzezinska‐Blaszczyk and A. Pietrzak , “Tumor Necrosis Factor Alpha (TNF‐Alpha) Activates Human Adenoidal and Cutaneous Mast Cells to Histamine Secretion,” Immunology Letters 59, no. 3 (1997): 139–143, 10.1016/s0165-2478(97)00115-6. 9419020

[55] W. R. Coward , Y. Okayama , H. Sagara , S. J. Wilson , S. T. Holgate , and M. K. Church , “NF‐κB and TNF‐α: A Positive Autocrine Loop in Human Lung Mast Cells?,” Journal of Immunology 169, no. 9 (2002): 5287–5293, 10.4049/jimmunol.169.9.5287. 12391248

