# Supplementary material for: Identifying Genetic Variants in Patients With Cefaclor‐Induced Anaphylaxis Using Human Leukocyte Antigen Typing and Whole‐Exome Sequencing
Source: Clin Transl Allergy. 2025 Sep 20;15(9):e70103. doi: 10.1002/clt2.70103 (PMC12449841; doi:10.1002/clt2.70103)
Supplement: Supplementary file 2 — Figure S2: Distribution of HLA Class I and Class II genotypes in 41 tolerant controls. [file CLT2-15-e70103-s002.docx]

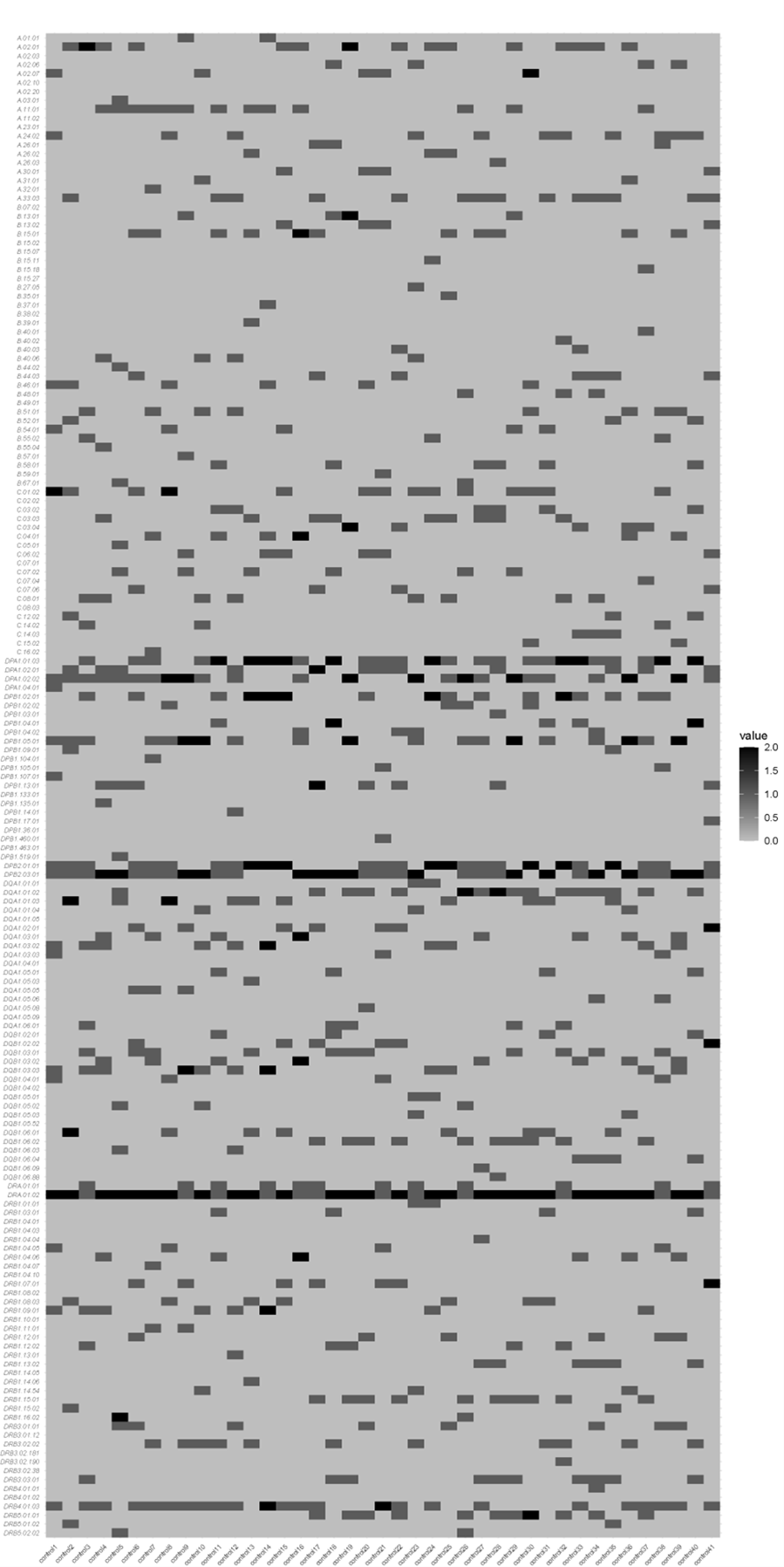


**Supplementary Figure E2.** **Distribution of HLA Class I and Class II genotypes in 41 tolerant controls.**

HLA genotypes were identified by next-generation sequencing (NGS) in 41 tolerant controls. Frequencies of Class I and Class II alleles are shown.
