# Supplementary material for: Identifying Genetic Variants in Patients With Cefaclor‐Induced Anaphylaxis Using Human Leukocyte Antigen Typing and Whole‐Exome Sequencing
Source: Clin Transl Allergy. 2025 Sep 20;15(9):e70103. doi: 10.1002/clt2.70103 (PMC12449841; doi:10.1002/clt2.70103)
Supplement: Supplementary file 3 — Table S1: Demographic and baseline clinical characteristics of patients with cefaclor‐induced anaphylaxis and tolerant controls. [file CLT2-15-e70103-s005.docx]

**Supplementary table E1.** Demographic and baseline clinical characteristics of patients with cefaclor-induced anaphylaxis and tolerant controls

| **Variables** | **Cefaclor-induced anaphylaxis (N = 33)** | **Control**  **(N = 41)** |
| --- | --- | --- |
| **Sex (female)** | 26 (78.8 %) | 32 (78.0 %) |
| **Age (years)** | 46.7 ± 12.3 | 35.4 ± 8.1**^*^** |
| **BMI (kg/m^2^)** | 24.3 ± 4.6 | 22.22 ± 3.38 |
| **Underlying Diseases** |  |  |
| Asthma | 1 (3.0 %) | 0 |
| Allergic rhinitis | 10 (30.3 %) | 10 (24.4 %) |
| Atopic dermatitis | 2 (6.1 %) | 1 (2.4 %) |
| Chronic urticaria | 3 (9.1 %) | 2 (4.9 %) |
| Drug allergy excluding cefaclor | 2 (6.1 %) | 0 |
| Food allergy**^*^** | 5 (15.2 %) | 0 |
| **Symptoms** |  |  |
| Cutaneous symptom | 31 (93.9 %) |  |
| Urticaria | 29 (87.9 %) |  |
| Angioedema | 17 (51.5 %) |  |
| Respiratory symptom | 22 (66.7 %) |  |
| Dyspnea | 19 (57.6 %) |  |
| Throat tightness | 6 (18.2 %) |  |
| Wheezing / Stridor | 2 (6.1 %) |  |
| Gastrointestinal symptom | 10 (30.3 %) |  |
| Nausea or vomiting | 3 (9.1 %) |  |
| Abdominal pain | 7 (21.2 %) |  |
| Diarrhea | 6 (18.2 %) |  |
| Cardiovascular symptom | 14 (42.4 %) |  |
| Hypotension | 11 (33.3 %) |  |
| Loss of consciousness | 8 (24.2 %) |  |
| Dizziness | 5 (15.2 %) |  |
| Tachycardia / palpitation | 2 (9.1 %) |  |
| Chest pain | 1 (3.0 %) |  |
| **Specific IgE to Cefaclor** |  |  |
| Number of Positives / Number tested | 32 / 32 |  |
| Specific IgE level, mean ± SD | 12.8 ± 20.3 |  |
| **Specific IgE to Beta-Lactam Antibiotics** |  |  |
| Amoxicilloyl (N _positive_ / N _tested_) | 0 / 17 |  |
| Ampicilloyl (N _positive_ / N _tested_) | 2 / 10 |  |
| Penicilloyl G (N _positive_ / N _tested_) | 2 / 27 |  |
| Penicilloyl V (N _positive_ / N _tested_) | 5 / 21 |  |

Values are presented as number (%) or mean ± standard deviation.

*P < 0.05 indicates statistically significant differences between groups.
