# Supplementary material for: Identifying Genetic Variants in Patients With Cefaclor‐Induced Anaphylaxis Using Human Leukocyte Antigen Typing and Whole‐Exome Sequencing
Source: Clin Transl Allergy. 2025 Sep 20;15(9):e70103. doi: 10.1002/clt2.70103 (PMC12449841; doi:10.1002/clt2.70103)
Supplement: Supplementary file 4 — Table S2: One hundred sixty‐four candidate variants associated with cefaclor‐induced anaphylaxis identified by exome‐wide association study. [file CLT2-15-e70103-s003.docx]

**Supplementary table E2.** 164 candidate variants associated with cefaclor-induced anaphylaxis identified by exome-wide association study

| CHR | Position | Variant | Gene | REF | RA | Allele frequency (2N) | | Statistics | | | | |  |  |
| --- | --- | --- | --- | --- | --- | --- | --- | --- | --- | --- | --- | --- | --- | --- |
|  |  |  |  |  |  | Patient (N=33) | Control (N=41) | Odds Ratio | 95% CI (lower) | 95% CI (upper) | **P*-value | *FDR adjusted  *P*-value | SIFT^a^ | Polyphen2^b^ |
| 1 | 1588744 | rs79724854 | CDK11B | AGCG | A | 0.424 | 0.073 | 47.607 | 10.783 | 210.179 | 3.42E-07 | 0.001244 |  |  |
| 1 | 1599888 | rs2072923 | CDK11B | A | G | 0.561 | 0.073 | 81.904 | 14.682 | 456.904 | 5.08E-07 | 0.001567 |  |  |
| 1 | 1654065 | rs35174499 | CDK11B | AGCG | A | 0.788 | 0.134 | 8.580 | 3.669 | 20.063 | 7.08E-07 | 0.001791 |  |  |
| 1 | 13448547 | rs200801174 | PRAMEF13 | T | C | 0.864 | 0.085 | 13.214 | 5.113 | 34.151 | 9.92E-08 | 0.000798 | T | B |
| 1 | 13448548 | rs201453721 | PRAMEF13 | G | A | 0.864 | 0.085 | 13.214 | 5.113 | 34.151 | 9.92E-08 | 0.000798 | T | B |
| 1 | 13448551 | rs199771678 | PRAMEF13 | T | C | 0.864 | 0.085 | 13.214 | 5.113 | 34.151 | 9.92E-08 | 0.000798 | T | B |
| 1 | 16386305 | rs372070031 | CLCNKB | G | GC | 0.667 | 0.098 | 14.536 | 4.839 | 43.669 | 1.85E-06 | 0.003434 |  |  |
| 1 | 16890671 | rs2419526 | NBPF1 | T | C | 0.515 | 0.085 | 183.959 | 19.922 | 1698.669 | 4.27E-06 | 0.005891 |  |  |
| 1 | 16890672 | rs2419525 | NBPF1 | G | A | 0.515 | 0.085 | 183.959 | 19.922 | 1698.669 | 4.27E-06 | 0.005891 |  |  |
| 1 | 16976193 | rs61772256 | MST1P2 | C | T | 0.561 | 0.098 | 18.639 | 5.561 | 62.471 | 2.13E-06 | 0.003598 |  |  |
| 1 | 17084536 | rs142741624 | MST1L | TGGAACA | T | 0.439 | 0.073 | 43.084 | 10.973 | 169.167 | 6.94E-08 | 0.000798 |  |  |
| 1 | 121129557 | rs200881439 | SRGAP2B | T | C | 0.333 | 0.024 | 38.999 | 7.915 | 192.159 | 6.72E-06 | 0.008137 |  |  |
| 1 | 143401063 | rs61799775 | LOC102723769-MIR6077 | T | G | 0.561 | 0.073 | 19.077 | 5.533 | 65.771 | 3.02E-06 | 0.004594 |  |  |
| 1 | 144811810 |  | NBPF20 | A | G | 0.470 | 0.061 | 194.096 | 26.440 | 1424.872 | 2.22E-07 | 0.000933 | T |  |
| 1 | 145368432 |  | NBPF20 | C | A | 0.364 | 0.061 | 25.928 | 6.529 | 102.960 | 3.72E-06 | 0.005354 | T |  |
| 1 | 152190682 | rs71625163 | HRNR | G | A | 0.667 | 0.085 | 12.067 | 4.219 | 34.514 | 3.40E-06 | 0.004993 |  |  |
| 1 | 152278662 |  | FLG | A | G | 0.348 | 0.061 | 17.119 | 5.102 | 57.436 | 4.25E-06 | 0.005891 |  |  |
| 1 | 219783709 | rs368783050 | LOC102723886-RNU5F-1 | G | A | 0.530 | 0.061 | 21.024 | 5.916 | 74.718 | 2.51E-06 | 0.004025 |  |  |
| 2 | 89103870 |  | ANKRD36BP2 | A | AT | 0.348 | 0.037 | 29.453 | 7.296 | 118.904 | 2.02E-06 | 0.003450 |  |  |
| 2 | 89104896 | rs5832744 | ANKRD36BP2 | C | CTG | 0.455 | 0.073 | 65.862 | 14.229 | 304.866 | 8.49E-08 | 0.000798 |  |  |
| 2 | 89104897 | rs62996860 | ANKRD36BP2 | CA | C | 0.455 | 0.073 | 65.862 | 14.229 | 304.866 | 8.49E-08 | 0.000798 |  |  |
| 2 | 91924935 |  | LOC101927050-GGT8P | C | T | 0.470 | 0.073 | 127.609 | 20.538 | 792.865 | 1.96E-07 | 0.000886 |  |  |
| 2 | 96517471 | rs77124870 | ANKRD36C | A | G | 0.485 | 0.073 | 260.423 | 26.323 | 2576.445 | 1.97E-06 | 0.003434 | T |  |
| 2 | 96519558 | rs373126569 | ANKRD36C | ATCGT | A | 0.424 | 0.073 | 37.967 | 9.681 | 148.896 | 1.83E-07 | 0.000886 |  |  |
| 2 | 96519611 | rs371319997 | ANKRD36C | TGATAA | T | 0.439 | 0.049 | 98.964 | 17.646 | 555.035 | 1.76E-07 | 0.000886 |  |  |
| 2 | 96521486 | rs201100797 | ANKRD36C | G | A | 0.470 | 0.061 | 134.886 | 22.075 | 824.205 | 1.09E-07 | 0.000798 | T |  |
| 2 | 96594777 | rs78943121 | ANKRD36C | C | T | 0.439 | 0.049 | 73.556 | 15.943 | 339.370 | 3.60E-08 | 0.000798 |  |  |
| 2 | 96610495 | rs144198449 | ANKRD36C | G | A | 0.379 | 0.049 | 34.272 | 8.415 | 139.573 | 8.10E-07 | 0.001950 |  |  |
| 2 | 96610805 | rs371613132 | ANKRD36C | AAGAAAG | A | 0.470 | 0.061 | 134.886 | 22.075 | 824.205 | 1.09E-07 | 0.000798 |  |  |
| 2 | 97845567 |  | ANKRD36 | TATTG | T | 0.485 | 0.073 | 260.423 | 26.323 | 2576.445 | 1.97E-06 | 0.003434 |  |  |
| 2 | 97845580 |  | ANKRD36 | G | GT | 0.485 | 0.073 | 260.423 | 26.323 | 2576.445 | 1.97E-06 | 0.003434 |  |  |
| 2 | 97845723 |  | ANKRD36 | T | G | 0.379 | 0.061 | 22.845 | 6.637 | 78.640 | 7.02E-07 | 0.001791 |  |  |
| 2 | 97851073 |  | ANKRD36 | GC | G | 0.439 | 0.073 | 48.466 | 11.604 | 202.432 | 1.03E-07 | 0.000798 |  |  |
| 2 | 97851078 |  | ANKRD36 | A | AG | 0.455 | 0.073 | 65.862 | 14.229 | 304.866 | 8.49E-08 | 0.000798 |  |  |
| 2 | 97851082 |  | ANKRD36 | GTAAT | G | 0.455 | 0.073 | 65.862 | 14.229 | 304.866 | 8.49E-08 | 0.000798 |  |  |
| 2 | 97909803 | rs374065576 | ANKRD36 | CTT | C | 0.515 | 0.073 | 241.500 | 24.444 | 2385.931 | 2.66E-06 | 0.004144 |  |  |
| 2 | 130832292 | rs75775141 | POTEF | T | A | 0.712 | 0.122 | 14.893 | 4.859 | 45.651 | 2.29E-06 | 0.003832 | D | B |
| 2 | 130832358 | rs201946437 | POTEF | T | C | 0.742 | 0.122 | 9.898 | 3.951 | 24.800 | 9.99E-07 | 0.002241 | T | B |
| 2 | 131377707 | rs201225245 | POTEJ | A | G | 0.970 | 0.146 | 21.791 | 5.738 | 82.757 | 6.01E-06 | 0.007463 |  |  |
| 2 | 132022031 |  | POTEE | C | T | 0.424 | 0.061 | 42.754 | 10.882 | 167.981 | 7.49E-08 | 0.000798 |  |  |
| 3 | 75718199 | rs141799937 | FRG2EP | C | T | 0.485 | 0.073 | 260.423 | 26.323 | 2576.445 | 1.97E-06 | 0.003434 |  |  |
| 3 | 100170600 | rs71132521 | LNP1 | A | ATCCTAGAAGGCATTCTCAT GAGGACCAGGAATTCCGATG CCGATCGTCTGACCGTCT | 0.712 | 0.110 | 13.079 | 4.528 | 37.777 | 2.03E-06 | 0.003450 |  |  |
| 3 | 161147239 |  | LOC101243545 | C | G | 0.364 | 0.024 | 52.845 | 10.430 | 267.731 | 1.65E-06 | 0.003358 |  |  |
| 3 | 161147240 |  | LOC101243545 | G | A | 0.364 | 0.024 | 52.845 | 10.430 | 267.731 | 1.65E-06 | 0.003358 |  |  |
| 3 | 195453406 | rs3210209 | MUC20 | G | C | 0.379 | 0.061 | 22.845 | 6.637 | 78.640 | 7.02E-07 | 0.001791 |  |  |
| 3 | 195453412 | rs1063306 | MUC20 | C | T | 0.379 | 0.061 | 22.845 | 6.637 | 78.640 | 7.02E-07 | 0.001791 |  |  |
| 3 | 195507475 | rs779277004 | MUC4 | G | C | 0.379 | 0.049 | 45.870 | 9.236 | 227.811 | 2.89E-06 | 0.004420 | D | P |
| 3 | 195508005 | rs79196348 | MUC4 | A | G | 0.394 | 0.049 | 35.114 | 9.202 | 133.993 | 1.91E-07 | 0.000886 |  |  |
| 3 | 195508010 | rs61388923 | MUC4 | C | A | 0.394 | 0.049 | 35.114 | 9.202 | 133.993 | 1.91E-07 | 0.000886 | D | D |
| 3 | 195513398 |  | MUC4 | C | CGGTGACAGGAAGAGGGGTG GCATGACCTGTGGATACTGA GGAATTGTCGGTGACAGGAA GAGGGGTGGCGTGACCGGTG GATGCTGAGGAAGTGCT | 0.333 | 0.049 | 19.903 | 5.437 | 72.859 | 6.26E-06 | 0.007633 |  |  |
| 4 | 4239587 | rs62286915 | TMEM128 | G | T | 0.470 | 0.073 | 127.609 | 20.538 | 792.865 | 1.96E-07 | 0.000886 |  |  |
| 4 | 151177340 | rs13152819 | DCLK2 | C | T | 0.682 | 0.122 | 5.714 | 2.708 | 12.057 | 4.76E-06 | 0.006305 | T | B |
| 5 | 796064 | rs144616948 | ZDHHC11 | T | TCAGTACTGTATGCCCATTTCC | 0.545 | 0.061 | 38.326 | 8.879 | 165.422 | 1.03E-06 | 0.002274 |  |  |
| 5 | 796437 | rs140929317 | ZDHHC11 | T | A | 0.409 | 0.073 | 30.922 | 8.222 | 116.294 | 3.83E-07 | 0.001327 |  |  |
| 5 | 68862361 | rs375045774 | GTF2H2C | TG | T | 0.727 | 0.110 | 17.884 | 5.483 | 58.334 | 1.74E-06 | 0.003432 |  |  |
| 5 | 68931045 | rs199638022 | GUSBP3 | C | A | 0.409 | 0.049 | 42.102 | 10.746 | 164.959 | 7.96E-08 | 0.000798 |  |  |
| 5 | 69784815 |  | SMA5 | TCAAACA | T | 0.758 | 0.122 | 5.512 | 2.828 | 10.743 | 5.38E-07 | 0.001590 |  |  |
| 5 | 69784835 |  | SMA5 | T | C | 0.758 | 0.122 | 5.512 | 2.828 | 10.743 | 5.38E-07 | 0.001590 |  |  |
| 6 | 29857119 | rs200033654 | HLA-H | A | G | 0.379 | 0.061 | 22.845 | 6.637 | 78.640 | 7.02E-07 | 0.001791 |  |  |
| 6 | 29857128 | rs201917587 | HLA-H | C | T | 0.364 | 0.061 | 19.663 | 5.799 | 66.668 | 1.74E-06 | 0.003432 |  |  |
| 6 | 29857137 | rs200510382 | HLA-H | C | T | 0.348 | 0.061 | 17.119 | 5.102 | 57.436 | 4.25E-06 | 0.005891 |  |  |
| 6 | 29857138 | rs201223459 | HLA-H | A | G | 0.379 | 0.073 | 18.333 | 5.639 | 59.609 | 1.33E-06 | 0.002888 |  |  |
| 6 | 29857237 | rs150344284 | HLA-H | G | C | 0.485 | 0.073 | 15.316 | 4.743 | 49.460 | 5.05E-06 | 0.006609 |  |  |
| 6 | 29911799 |  | HLA-A | T | C | 0.379 | 0.073 | 19.410 | 5.798 | 64.971 | 1.50E-06 | 0.003151 |  |  |
| 6 | 29911806 |  | HLA-A | T | C | 0.394 | 0.073 | 25.852 | 7.076 | 94.445 | 8.65E-07 | 0.002009 |  |  |
| 6 | 32489852 | rs192498095 | HLA-DRB5 | A | G | 0.333 | 0.037 | 25.842 | 6.437 | 103.741 | 4.52E-06 | 0.006071 | D | P |
| 6 | 32551868 | rs200089806 | HLA-DRB1 | G | GC | 0.667 | 0.085 | 5.856 | 2.740 | 12.518 | 5.10E-06 | 0.006619 |  |  |
| 6 | 32557568 |  | HLA-DRB1 | C | CAT | 0.394 | 0.061 | 35.092 | 8.569 | 143.708 | 7.56E-07 | 0.001843 |  |  |
| 6 | 32557600 |  | HLA-DRB1 | T | TC | 0.333 | 0.049 | 19.903 | 5.437 | 72.859 | 6.26E-06 | 0.007633 |  |  |
| 6 | 33053736 |  | HLA-DPB1 | GGT | G | 0.545 | 0.049 | 23.296 | 6.275 | 86.492 | 2.55E-06 | 0.004028 |  |  |
| 6 | 33053740 | rs140608279 | HLA-DPB1 | T | TCA | 0.545 | 0.049 | 23.296 | 6.275 | 86.492 | 2.55E-06 | 0.004028 |  |  |
| 6 | 110797703 | rs150666553 | SLC22A16 | CCGCGGG | C | 0.636 | 0.073 | 26.879 | 7.216 | 100.113 | 9.31E-07 | 0.002112 |  |  |
| 6 | 110797713 |  | SLC22A16 | C | CCACCCCT | 0.636 | 0.073 | 26.879 | 7.216 | 100.113 | 9.31E-07 | 0.002112 |  |  |
| 7 | 100551390 | rs77795035 | MUC3A | T | A | 0.409 | 0.049 | 47.758 | 11.396 | 200.140 | 1.23E-07 | 0.000840 |  |  |
| 7 | 100551393 | rs79067082 | MUC3A | G | A | 0.409 | 0.049 | 47.758 | 11.396 | 200.140 | 1.23E-07 | 0.000840 |  |  |
| 7 | 100641872 | rs139851927 | MUC12 | T | C | 0.561 | 0.073 | 12.846 | 4.205 | 39.246 | 7.45E-06 | 0.008963 |  |  |
| 7 | 100647338 | rs199551523 | MUC12 | C | A | 0.636 | 0.098 | 7.063 | 3.038 | 16.424 | 5.61E-06 | 0.007096 | T |  |
| 7 | 100647339 | rs200522765 | MUC12 | G | A | 0.636 | 0.098 | 7.063 | 3.038 | 16.424 | 5.61E-06 | 0.007096 | T |  |
| 7 | 142119876 |  | TRY2P-MTRNR2L6 | CG | C | 0.455 | 0.073 | 65.862 | 14.229 | 304.866 | 8.49E-08 | 0.000798 |  |  |
| 7 | 142119879 |  | TRY2P-MTRNR2L6 | C | CCA | 0.455 | 0.073 | 65.862 | 14.229 | 304.866 | 8.49E-08 | 0.000798 |  |  |
| 7 | 142119881 |  | TRY2P-MTRNR2L6 | GA | G | 0.439 | 0.073 | 48.466 | 11.604 | 202.432 | 1.03E-07 | 0.000798 |  |  |
| 7 | 142131738 |  | TRY2P-MTRNR2L6 | G | GA | 0.470 | 0.073 | 90.573 | 17.004 | 482.436 | 1.29E-07 | 0.000850 |  |  |
| 7 | 142481996 |  | PRSS3P2 | G | GC | 0.803 | 0.098 | 35.663 | 7.656 | 166.123 | 5.29E-06 | 0.006829 |  |  |
| 7 | 144015687 | rs202096920 | OR2A42 | C | T | 0.803 | 0.134 | 12.820 | 4.706 | 34.924 | 6.07E-07 | 0.001713 | T |  |
| 7 | 144015720 | rs201568948 | OR2A42 | C | G | 0.970 | 0.146 | 16.608 | 5.139 | 53.669 | 2.67E-06 | 0.004144 | T |  |
| 8 | 11872992 | rs113737745 | DEFB134-DEFB130 | AGGT | A | 0.409 | 0.061 | 41.880 | 9.963 | 176.039 | 3.43E-07 | 0.001244 |  |  |
| 9 | 162405 | rs201338779 | CBWD1 | C | G | 0.712 | 0.085 | 11.894 | 4.369 | 32.377 | 1.26E-06 | 0.002763 |  |  |
| 9 | 39890309 | rs782697833 | SPATA31A1 | G | A | 0.955 | 0.146 | 14.636 | 5.087 | 42.105 | 6.45E-07 | 0.001791 | D | B |
| 9 | 68438589 | rs62543827 | FRG1JP | A | G | 0.424 | 0.073 | 37.967 | 9.681 | 148.896 | 1.83E-07 | 0.000886 |  |  |
| 9 | 69390020 | rs200769647 | ANKRD20A4 | T | A | 0.439 | 0.073 | 61.002 | 13.002 | 286.201 | 1.86E-07 | 0.000886 | T | B |
| 9 | 69391179 | . | ANKRD20A4 | CAT | C | 0.333 | 0.037 | 25.388 | 6.377 | 101.071 | 4.47E-06 | 0.006038 |  |  |
| 10 | 29578180 | rs71525544 | LYZL1 | A | ATGAAGCCAACTGTCTC | 0.803 | 0.122 | 11.387 | 4.490 | 28.881 | 3.01E-07 | 0.001166 |  |  |
| 10 | 51620278 | rs375387689 | TIMM23 | T | A | 0.652 | 0.098 | 48.711 | 9.377 | 253.038 | 3.79E-06 | 0.005424 |  |  |
| 10 | 51623602 |  | TIMM23 | C | G | 0.667 | 0.098 | 46.511 | 9.030 | 239.563 | 4.41E-06 | 0.006035 |  |  |
| 10 | 81270425 |  | EIF5AL1 | A | AG | 0.379 | 0.049 | 28.922 | 7.855 | 106.497 | 4.21E-07 | 0.001364 |  |  |
| 10 | 81343060 |  | SFTPA2-SFTPA1 | G | GCCAAGAACACATTATGGGC AGAGAGAAAGCTCACACTT | 0.515 | 0.073 | 13.424 | 4.366 | 41.273 | 5.84E-06 | 0.007343 |  |  |
| 11 | 1018229 | rs77630889 | MUC6 | C | G | 0.485 | 0.073 | 260.423 | 26.323 | 2576.445 | 1.97E-06 | 0.003434 |  |  |
| 11 | 1018523 | rs73403301 | MUC6 | A | G | 0.485 | 0.073 | 260.423 | 26.323 | 2576.445 | 1.97E-06 | 0.003434 |  |  |
| 11 | 48387647 | rs75615667 | OR4C45-OR4A47 | A | G | 0.409 | 0.061 | 34.871 | 9.231 | 131.734 | 1.63E-07 | 0.000886 | T |  |
| 11 | 56143424 | rs12788990 | OR8U8 | A | ACGGC | 0.455 | 0.061 | 88.612 | 17.384 | 451.697 | 6.81E-08 | 0.000798 | T | B |
| 11 | 56143425 |  | OR8U8 | TATCA | T | 0.470 | 0.061 | 134.886 | 22.075 | 824.205 | 1.09E-07 | 0.000798 |  |  |
| 11 | 71527696 | rs4021274 | ZNF705E | C | A | 0.485 | 0.073 | 14.452 | 4.613 | 45.270 | 4.55E-06 | 0.006071 |  |  |
| 12 | 9585598 | rs200479049 | DDX12P | T | C | 0.955 | 0.146 | 12.721 | 4.697 | 34.451 | 5.64E-07 | 0.001639 |  |  |
| 12 | 10586978 | rs144149096 | KLRC2 | A | AT | 0.500 | 0.085 | 185.661 | 20.155 | 1710.259 | 4.01E-06 | 0.005691 |  |  |
| 12 | 11420333 | rs3842295 | PRB3 | AG | A | 0.515 | 0.073 | 13.534 | 4.400 | 41.628 | 5.51E-06 | 0.007058 |  |  |
| 12 | 31256546 | rs1046457 | DDX11 | G | A | 0.803 | 0.110 | 10.997 | 4.373 | 27.654 | 3.46E-07 | 0.001244 | T | B |
| 12 | 40883781 | rs2588392 | MUC19 | G | A | 0.758 | 0.085 | 34.559 | 8.080 | 147.819 | 1.77E-06 | 0.003432 |  |  |
| 12 | 40883786 | rs2638877 | MUC19 | T | A | 0.758 | 0.085 | 34.559 | 8.080 | 147.819 | 1.77E-06 | 0.003432 |  |  |
| 12 | 40907227 | rs374317567 | MUC19 | CCAG | C | 0.439 | 0.061 | 65.352 | 14.113 | 302.626 | 9.04E-08 | 0.000798 |  |  |
| 12 | 40907233 | rs376023975 | MUC19 | ACT | A | 0.439 | 0.061 | 65.352 | 14.113 | 302.626 | 9.04E-08 | 0.000798 |  |  |
| 12 | 40907236 | rs371287727 | MUC19 | ACTGGCCTT | A | 0.424 | 0.061 | 51.309 | 11.746 | 224.118 | 1.65E-07 | 0.000886 |  |  |
| 13 | 19999954 | rs200353856 | TPTE2 | T | TGCGAA | 0.758 | 0.098 | 38.238 | 7.876 | 185.649 | 6.18E-06 | 0.007630 |  |  |
| 14 | 19807080 | rs59504060 | DUXAP10-LINC01296 | CA | C | 0.500 | 0.073 | 93.681 | 16.756 | 523.770 | 2.34E-07 | 0.000944 |  |  |
| 14 | 65068330 | rs372346293 | PPP1R36-PLEKHG3 | CTG | C | 0.621 | 0.085 | 19.690 | 5.620 | 68.980 | 3.18E-06 | 0.004756 |  |  |
| 14 | 65068341 | rs377267839 | PPP1R36-PLEKHG3 | TTCTC | T | 0.621 | 0.085 | 19.690 | 5.620 | 68.980 | 3.18E-06 | 0.004756 |  |  |
| 15 | 20456665 | rs8026258 | CHR_START-CHEK2P2 | T | A | 0.439 | 0.073 | 48.466 | 11.604 | 202.432 | 1.03E-07 | 0.000798 |  |  |
| 15 | 20456673 | rs8033709 | CHR_START-CHEK2P2 | C | G | 0.439 | 0.073 | 48.466 | 11.604 | 202.432 | 1.03E-07 | 0.000798 |  |  |
| 15 | 20456677 | rs8037872 | CHR_START-CHEK2P2 | G | A | 0.439 | 0.073 | 48.466 | 11.604 | 202.432 | 1.03E-07 | 0.000798 |  |  |
| 15 | 23265262 |  | GOLGA8IP | C | T | 0.697 | 0.037 | 30.951 | 7.147 | 134.030 | 4.43E-06 | 0.006035 |  |  |
| 15 | 30436776 | rs201209881 | GOLGA8T | G | A | 0.470 | 0.098 | 17.990 | 5.467 | 59.202 | 1.98E-06 | 0.003434 |  |  |
| 15 | 31092846 | rs34007223 | LOC100288637-HERC2P10 | TC | T | 0.348 | 0.049 | 21.337 | 5.978 | 76.156 | 2.42E-06 | 0.003976 |  |  |
| 15 | 76078004 | rs34818937 | MIR4313-UBE2Q2 | G | GT | 0.515 | 0.073 | 73.176 | 14.159 | 378.175 | 3.01E-07 | 0.001166 |  |  |
| 16 | 67592 | rs201747478 | DDX11L10 | C | T | 0.348 | 0.024 | 46.175 | 9.157 | 232.843 | 3.44E-06 | 0.004993 |  |  |
| 16 | 67593 | rs199775480 | DDX11L10 | A | G | 0.348 | 0.024 | 46.175 | 9.157 | 232.843 | 3.44E-06 | 0.004993 |  |  |
| 16 | 67604 | rs4021659 | DDX11L10 | T | C | 0.348 | 0.024 | 46.175 | 9.157 | 232.843 | 3.44E-06 | 0.004993 |  |  |
| 16 | 1291608 | rs201820654 | TPSAB1 | A | G | 0.409 | 0.049 | 63.748 | 12.370 | 328.527 | 6.82E-07 | 0.001791 | T | B |
| 16 | 1291623 | rs765144578 | TPSAB1 | C | T | 0.439 | 0.049 | 98.964 | 17.646 | 555.035 | 1.76E-07 | 0.000886 | D | B |
| 16 | 1291717 | rs202230226 | TPSAB1 | T | TG | 0.455 | 0.061 | 126.609 | 20.370 | 786.921 | 2.07E-07 | 0.000886 |  |  |
| 16 | 1291800 | rs375717182 | TPSAB1 | G | C | 0.455 | 0.061 | 126.609 | 20.370 | 786.921 | 2.07E-07 | 0.000886 |  |  |
| 16 | 1291850 | rs200171211 | TPSAB1 | T | C | 0.455 | 0.061 | 126.609 | 20.370 | 786.921 | 2.07E-07 | 0.000886 |  |  |
| 16 | 1291941 | rs201280504 | TPSAB1 | G | A | 0.455 | 0.061 | 89.134 | 15.677 | 506.774 | 4.11E-07 | 0.001364 | T | B |
| 16 | 2088405 | rs149481694 | NTHL1 | CCCCCCTTCCCCT | C | 0.621 | 0.073 | 10.746 | 3.879 | 29.766 | 4.93E-06 | 0.006489 |  |  |
| 16 | 32442104 | rs139974249 | LOC390705-TP53TG3E | ATAT | A | 0.394 | 0.061 | 35.092 | 8.569 | 143.708 | 7.56E-07 | 0.001843 |  |  |
| 16 | 32936832 |  | SLC6A10P-TP53TG3 | A | G | 0.394 | 0.049 | 35.114 | 9.202 | 133.993 | 1.91E-07 | 0.000886 |  |  |
| 16 | 32936837 |  | SLC6A10P-TP53TG3 | G | T | 0.379 | 0.049 | 28.922 | 7.855 | 106.497 | 4.21E-07 | 0.001364 |  |  |
| 16 | 33424917 | rs1906318 | LOC390705-ENPP7P13 | A | C | 0.485 | 0.073 | 260.423 | 26.323 | 2576.445 | 1.97E-06 | 0.003434 |  |  |
| 16 | 33424950 | rs2927971 | LOC390705-ENPP7P13 | C | T | 0.485 | 0.073 | 260.423 | 26.323 | 2576.445 | 1.97E-06 | 0.003434 |  |  |
| 16 | 33500900 | rs4079165 | LOC390705-ENPP7P13 | C | T | 0.379 | 0.049 | 29.893 | 7.974 | 112.065 | 4.67E-07 | 0.001464 |  |  |
| 16 | 33500915 | rs4079164 | LOC390705-ENPP7P13 | T | A | 0.379 | 0.049 | 29.893 | 7.974 | 112.065 | 4.67E-07 | 0.001464 |  |  |
| 16 | 69996596 | rs372963769 | CLEC18A | G | A | 0.848 | 0.110 | 19.400 | 6.025 | 62.461 | 6.68E-07 | 0.001791 |  |  |
| 16 | 74425278 | rs7195896 | NPIPB15 | T | C | 0.364 | 0.049 | 29.783 | 7.382 | 120.155 | 1.85E-06 | 0.003434 |  |  |
| 16 | 90161902 | rs6500471 | PRDM7-FAM157C | A | G | 0.864 | 0.122 | 16.600 | 5.623 | 49.008 | 3.65E-07 | 0.001288 |  |  |
| 17 | 14095639 | rs373313742 | COX10 | A | G | 0.439 | 0.049 | 98.964 | 17.646 | 555.035 | 1.76E-07 | 0.000886 |  |  |
| 17 | 15639050 |  | TBC1D26 | G | GT | 0.424 | 0.024 | 55.333 | 10.733 | 285.259 | 1.62E-06 | 0.003358 |  |  |
| 17 | 33680807 | rs9897352 | SLFN11 | G | A | 0.970 | 0.146 | 21.791 | 5.738 | 82.757 | 6.01E-06 | 0.007463 |  |  |
| 18 | 14183885 |  | ANKRD20A5P | A | AT | 0.485 | 0.061 | 396.254 | 34.974 | 4489.471 | 1.37E-06 | 0.002898 |  |  |
| 19 | 1619749 | rs10648013 | TCF3 | A | AGGGTG | 0.712 | 0.122 | 11.336 | 4.198 | 30.612 | 1.67E-06 | 0.003358 |  |  |
| 19 | 43860192 | rs71337594 | CD177 | T | G | 0.939 | 0.146 | 14.747 | 5.212 | 41.726 | 3.95E-07 | 0.001346 | T |  |
| 19 | 43860251 | rs199668750 | CD177 | G | A | 0.879 | 0.110 | 18.859 | 5.859 | 60.697 | 8.46E-07 | 0.001988 | T |  |
| 19 | 43860255 | rs200662237 | CD177 | T | G | 0.879 | 0.110 | 18.859 | 5.859 | 60.697 | 8.46E-07 | 0.001988 | T |  |
| 19 | 43865319 |  | CD177 | CCT | C | 0.606 | 0.110 | 21.396 | 5.983 | 76.512 | 2.46E-06 | 0.003976 |  |  |
| 19 | 43865324 |  | CD177 | A | AAG | 0.606 | 0.110 | 21.396 | 5.983 | 76.512 | 2.46E-06 | 0.003976 |  |  |
| 19 | 55286770 |  | KIR2DL1 | C | G | 0.424 | 0.073 | 47.607 | 10.783 | 210.179 | 3.42E-07 | 0.001244 | T | B |
| 20 | 26061876 |  | FAM182A | G | GAA | 0.439 | 0.073 | 48.466 | 11.604 | 202.432 | 1.03E-07 | 0.000798 |  |  |
| 20 | 26061878 |  | FAM182A | GGATC | G | 0.439 | 0.073 | 48.466 | 11.604 | 202.432 | 1.03E-07 | 0.000798 |  |  |
| 20 | 29623113 | rs150951095 | FRG1BP | CA | C | 0.485 | 0.061 | 273.140 | 28.054 | 2659.378 | 1.36E-06 | 0.002898 |  |  |
| 21 | 14982746 | rs369746921 | POTED | G | A | 0.379 | 0.049 | 45.870 | 9.236 | 227.811 | 2.89E-06 | 0.004420 | T | B |
| 21 | 15440935 | rs201093426 | ANKRD20A11P-LIPI | G | A | 0.424 | 0.049 | 37.043 | 8.968 | 153.017 | 6.01E-07 | 0.001713 |  |  |
| 22 | 16968390 | rs371730763 | OR11H1-CCT8L2 | AGAGTTTGCCAACCCAGACT  TTATGGAATCCATCTT | A | 0.455 | 0.061 | 88.612 | 17.384 | 451.697 | 6.81E-08 | 0.000798 |  |  |
| 22 | 18775061 | rs189425726 | GGT3P | G | A | 0.485 | 0.073 | 203.961 | 22.431 | 1854.565 | 2.34E-06 | 0.003881 |  |  |
| 22 | 21576356 | rs201958718 | FAM230B-POM121L8P | T | G | 0.500 | 0.073 | 93.681 | 16.756 | 523.770 | 2.34E-07 | 0.000944 |  |  |
| 22 | 23248851 | rs71797740 | IGLL5-RSPH14 | CA | C | 0.394 | 0.073 | 21.702 | 6.514 | 72.308 | 5.40E-07 | 0.001590 |  |  |
| 22 | 25003852 | rs3033885 | GGT1 | GGC | G | 0.485 | 0.073 | 260.423 | 26.323 | 2576.445 | 1.97E-06 | 0.003434 |  |  |
| 22 | 25023868 |  | GGT1 | GA | G | 0.394 | 0.061 | 35.092 | 8.569 | 143.708 | 7.56E-07 | 0.001843 |  |  |

CHR, chromosome; REF, reference allele; RA, risk allele; CI, confidence interval; FDR, false discovery rate; SIFT, Sorting Intolerant From Tolerant; PolyPhen-2, Polymorphism Phenotyping v2.

*P value from logistic regression adjusted for sex, comparing 33 patients with cefaclor-induced anaphylaxis and 41 tolerant controls.

^a^Functional impact predicted by SIFT.

^b^Functional impact predicted by PolyPhen-2.
