# Supplementary material for: Identifying Genetic Variants in Patients With Cefaclor‐Induced Anaphylaxis Using Human Leukocyte Antigen Typing and Whole‐Exome Sequencing
Source: Clin Transl Allergy. 2025 Sep 20;15(9):e70103. doi: 10.1002/clt2.70103 (PMC12449841; doi:10.1002/clt2.70103)
Supplement: Supplementary file 5 — Table S3: Detailed gene lists and enrichment scores for the TNF‐α signaling via NF‐κB (M5890) pathway. [file CLT2-15-e70103-s004.docx]

**Supplementary table E3.** Detailed gene lists and enrichment scores for the TNF-α signaling via NF-κB (M5890) pathway

| **Gene symbol** | **Gene name** | **Rank in gene list** | **Rank metric score** | **Running enrichment score** | **Core enrichment** |
| --- | --- | --- | --- | --- | --- |
| G0S2 | G0/G1 switch 2 [Source:HGNC Symbol;Acc:HGNC:30229] | 138 | 0.993 | 0.0017 | Yes |
| BHLHE40 | basic helix-loop-helix family member e40 [Source:HGNC Symbol;Acc:HGNC:1046] | 147 | 0.992 | 0.01 | Yes |
| TNFRSF9 | TNF receptor superfamily member 9 [Source:HGNC Symbol;Acc:HGNC:11924] | 284 | 0.992 | 0.0119 | Yes |
| PLPP3 | phospholipid phosphatase 3 [Source:HGNC Symbol;Acc:HGNC:9229] | 400 | 0.992 | 0.0148 | Yes |
| LIF | LIF interleukin 6 family cytokine [Source:HGNC Symbol;Acc:HGNC:6596] | 565 | 0.992 | 0.0152 | Yes |
| CXCL3 | C-X-C motif chemokine ligand 3 [Source:HGNC Symbol;Acc:HGNC:4604] | 602 | 0.992 | 0.0221 | Yes |
| F2RL1 | F2R like trypsin receptor 1 [Source:HGNC Symbol;Acc:HGNC:3538] | 849 | 0.992 | 0.0184 | Yes |
| MARCKS | myristoylated alanine rich protein kinase C substrate [Source:HGNC Symbol;Acc:HGNC:6759] | 881 | 0.992 | 0.0255 | Yes |
| DNAJB4 | DnaJ heat shock protein family (Hsp40) member B4 [Source:HGNC Symbol;Acc:HGNC:14886] | 1026 | 0.992 | 0.0269 | Yes |
| JUNB | JunB proto-oncogene, AP-1 transcription factor subunit [Source:HGNC Symbol;Acc:HGNC:6205] | 1144 | 0.992 | 0.0297 | Yes |
| RNF19B | ring finger protein 19B [Source:HGNC Symbol;Acc:HGNC:26886] | 1333 | 0.991 | 0.029 | Yes |
| ID2 | inhibitor of DNA binding 2 [Source:HGNC Symbol;Acc:HGNC:5361] | 1429 | 0.991 | 0.0329 | Yes |
| DUSP1 | dual specificity phosphatase 1 [Source:HGNC Symbol;Acc:HGNC:3064] | 1469 | 0.991 | 0.0396 | Yes |
| MSC | musculin [Source:HGNC Symbol;Acc:HGNC:7321] | 1535 | 0.991 | 0.045 | Yes |
| CXCL6 | C-X-C motif chemokine ligand 6 [Source:HGNC Symbol;Acc:HGNC:10643] | 1568 | 0.991 | 0.0521 | Yes |
| TIPARP | TCDD inducible poly(ADP-ribose) polymerase [Source:HGNC Symbol;Acc:HGNC:23696] | 1571 | 0.991 | 0.0606 | Yes |
| YRDC | yrdC N6-threonylcarbamoyltransferase domain containing [Source:HGNC Symbol;Acc:HGNC:28905] | 1578 | 0.991 | 0.069 | Yes |
| MAP3K8 | mitogen-activated protein kinase kinase kinase 8 [Source:HGNC Symbol;Acc:HGNC:6860] | 1688 | 0.991 | 0.0722 | Yes |
| DENND5A | DENN domain containing 5A [Source:HGNC Symbol;Acc:HGNC:19344] | 1705 | 0.991 | 0.0801 | Yes |
| BTG2 | BTG anti-proliferation factor 2 [Source:HGNC Symbol;Acc:HGNC:1131] | 1714 | 0.991 | 0.0884 | Yes |
| IL18 | interleukin 18 [Source:HGNC Symbol;Acc:HGNC:5986] | 1794 | 0.991 | 0.0931 | Yes |
| BIRC2 | baculoviral IAP repeat containing 2 [Source:HGNC Symbol;Acc:HGNC:590] | 2083 | 0.991 | 0.0873 | Yes |
| CCN1 | cellular communication network factor 1 [Source:HGNC Symbol;Acc:HGNC:2654] | 2089 | 0.991 | 0.0957 | Yes |
| ATP2B1 | ATPase plasma membrane Ca2+ transporting 1 [Source:HGNC Symbol;Acc:HGNC:814] | 2278 | 0.991 | 0.0949 | Yes |
| TNFAIP2 | TNF alpha induced protein 2 [Source:HGNC Symbol;Acc:HGNC:11895] | 2422 | 0.991 | 0.0964 | Yes |
| EGR1 | early growth response 1 [Source:HGNC Symbol;Acc:HGNC:3238] | 2453 | 0.991 | 0.1036 | Yes |
| CD69 | CD69 molecule [Source:HGNC Symbol;Acc:HGNC:1694] | 2487 | 0.991 | 0.1106 | Yes |
| FOSB | FosB proto-oncogene, AP-1 transcription factor subunit [Source:HGNC Symbol;Acc:HGNC:3797] | 2549 | 0.991 | 0.1162 | Yes |
| EGR3 | early growth response 3 [Source:HGNC Symbol;Acc:HGNC:3240] | 2651 | 0.991 | 0.1198 | Yes |
| EHD1 | EH domain containing 1 [Source:HGNC Symbol;Acc:HGNC:3242] | 2657 | 0.991 | 0.1282 | Yes |
| FOS | Fos proto-oncogene, AP-1 transcription factor subunit [Source:HGNC Symbol;Acc:HGNC:3796] | 2861 | 0.991 | 0.1267 | Yes |
| PANX1 | pannexin 1 [Source:HGNC Symbol;Acc:HGNC:8599] | 2866 | 0.991 | 0.1352 | Yes |
| BCL2A1 | BCL2 related protein A1 [Source:HGNC Symbol;Acc:HGNC:991] | 2935 | 0.991 | 0.1404 | Yes |
| TSC22D1 | TSC22 domain family member 1 [Source:HGNC Symbol;Acc:HGNC:16826] | 2969 | 0.991 | 0.1474 | Yes |
| RIPK2 | receptor interacting serine/threonine kinase 2 [Source:HGNC Symbol;Acc:HGNC:10020] | 2987 | 0.99 | 0.1552 | Yes |
| KLF10 | KLF transcription factor 10 [Source:HGNC Symbol;Acc:HGNC:11810] | 3004 | 0.99 | 0.1631 | Yes |
| FJX1 | four-jointed box kinase 1 [Source:HGNC Symbol;Acc:HGNC:17166] | 3232 | 0.99 | 0.1604 | Yes |
| DRAM1 | DNA damage regulated autophagy modulator 1 [Source:HGNC Symbol;Acc:HGNC:25645] | 3240 | 0.99 | 0.1687 | Yes |
| CEBPB | CCAAT enhancer binding protein beta [Source:HGNC Symbol;Acc:HGNC:1834] | 3349 | 0.99 | 0.1719 | Yes |
| CSF1 | colony stimulating factor 1 [Source:HGNC Symbol;Acc:HGNC:2432] | 3465 | 0.99 | 0.1748 | Yes |
| MYC | MYC proto-oncogene, bHLH transcription factor [Source:HGNC Symbol;Acc:HGNC:7553] | 3563 | 0.99 | 0.1786 | Yes |
| PER1 | period circadian regulator 1 [Source:HGNC Symbol;Acc:HGNC:8845] | 3683 | 0.989 | 0.1813 | Yes |
| AREG | amphiregulin [Source:HGNC Symbol;Acc:HGNC:651] | 3929 | 0.989 | 0.1776 | Yes |
| MAP2K3 | mitogen-activated protein kinase kinase 3 [Source:HGNC Symbol;Acc:HGNC:6843] | 4009 | 0.989 | 0.1823 | Yes |
| TRIB1 | tribbles pseudokinase 1 [Source:HGNC Symbol;Acc:HGNC:16891] | 4049 | 0.989 | 0.189 | Yes |
| FOSL2 | FOS like 2, AP-1 transcription factor subunit [Source:HGNC Symbol;Acc:HGNC:3798] | 4108 | 0.989 | 0.1947 | Yes |
| HBEGF | heparin binding EGF like growth factor [Source:HGNC Symbol;Acc:HGNC:3059] | 4183 | 0.989 | 0.1996 | Yes |
| F3 | coagulation factor III, tissue factor [Source:HGNC Symbol;Acc:HGNC:3541] | 4189 | 0.989 | 0.208 | Yes |
| FUT4 | fucosyltransferase 4 [Source:HGNC Symbol;Acc:HGNC:4015] | 4271 | 0.988 | 0.2126 | Yes |
| OLR1 | oxidized low density lipoprotein receptor 1 [Source:HGNC Symbol;Acc:HGNC:8133] | 4457 | 0.937 | 0.2115 | Yes |
| NINJ1 | ninjurin 1 [Source:HGNC Symbol;Acc:HGNC:7824] | 4461 | 0.937 | 0.2196 | Yes |
| NFKB2 | nuclear factor kappa B subunit 2 [Source:HGNC Symbol;Acc:HGNC:7795] | 4763 | 0.881 | 0.2121 | Yes |
| NFE2L2 | NFE2 like bZIP transcription factor 2 [Source:HGNC Symbol;Acc:HGNC:7782] | 4764 | 0.881 | 0.2199 | Yes |
| JUN | Jun proto-oncogene, AP-1 transcription factor subunit [Source:HGNC Symbol;Acc:HGNC:6204] | 4798 | 0.881 | 0.2259 | Yes |
| CXCL1 | C-X-C motif chemokine ligand 1 [Source:HGNC Symbol;Acc:HGNC:4602] | 4862 | 0.881 | 0.2305 | Yes |
| CLCF1 | cardiotrophin like cytokine factor 1 [Source:HGNC Symbol;Acc:HGNC:17412] | 4893 | 0.881 | 0.2367 | Yes |
| KLF9 | KLF transcription factor 9 [Source:HGNC Symbol;Acc:HGNC:1123] | 4943 | 0.881 | 0.2419 | Yes |
| TLR2 | toll like receptor 2 [Source:HGNC Symbol;Acc:HGNC:11848] | 5059 | 0.881 | 0.2439 | Yes |
| SLC16A6 | solute carrier family 16 member 6 [Source:HGNC Symbol;Acc:HGNC:10927] | 5147 | 0.881 | 0.2472 | Yes |
| BIRC3 | baculoviral IAP repeat containing 3 [Source:HGNC Symbol;Acc:HGNC:591] | 5228 | 0.881 | 0.2509 | Yes |
| CCL5 | C-C motif chemokine ligand 5 [Source:HGNC Symbol;Acc:HGNC:10632] | 5232 | 0.881 | 0.2585 | Yes |
| CCL2 | C-C motif chemokine ligand 2 [Source:HGNC Symbol;Acc:HGNC:10618] | 5234 | 0.881 | 0.2661 | Yes |
| ATF3 | activating transcription factor 3 [Source:HGNC Symbol;Acc:HGNC:785] | 5508 | 0.868 | 0.26 | Yes |
| PHLDA1 | pleckstrin homology like domain family A member 1 [Source:HGNC Symbol;Acc:HGNC:8933] | 5582 | 0.868 | 0.2639 | Yes |
| IFNGR2 | interferon gamma receptor 2 [Source:HGNC Symbol;Acc:HGNC:5440] | 5738 | 0.849 | 0.2636 | Yes |
| B4GALT5 | beta-1,4-galactosyltransferase 5 [Source:HGNC Symbol;Acc:HGNC:928] | 5789 | 0.84 | 0.2684 | Yes |
| DUSP5 | dual specificity phosphatase 5 [Source:HGNC Symbol;Acc:HGNC:3071] | 5810 | 0.837 | 0.2747 | Yes |
| IER3 | immediate early response 3 [Source:HGNC Symbol;Acc:HGNC:5392] | 5896 | 0.83 | 0.2777 | Yes |
| IL1B | interleukin 1 beta [Source:HGNC Symbol;Acc:HGNC:5992] | 5910 | 0.83 | 0.2843 | Yes |
| GCH1 | GTP cyclohydrolase 1 [Source:HGNC Symbol;Acc:HGNC:4193] | 6006 | 0.83 | 0.2868 | Yes |
| ACKR3 | atypical chemokine receptor 3 [Source:HGNC Symbol;Acc:HGNC:23692] | 6081 | 0.822 | 0.2903 | Yes |
| ZFP36 | ZFP36 ring finger protein [Source:HGNC Symbol;Acc:HGNC:12862] | 6140 | 0.822 | 0.2946 | Yes |
| RELA | RELA proto-oncogene, NF-kB subunit [Source:HGNC Symbol;Acc:HGNC:9955] | 6146 | 0.822 | 0.3015 | Yes |
| CXCL10 | C-X-C motif chemokine ligand 10 [Source:HGNC Symbol;Acc:HGNC:10637] | 6211 | 0.821 | 0.3055 | Yes |
| CXCL11 | C-X-C motif chemokine ligand 11 [Source:HGNC Symbol;Acc:HGNC:10638] | 6212 | 0.821 | 0.3127 | Yes |
| GPR183 | G protein-coupled receptor 183 [Source:HGNC Symbol;Acc:HGNC:3128] | 6286 | 0.813 | 0.3161 | Yes |
| CDKN1A | cyclin dependent kinase inhibitor 1A [Source:HGNC Symbol;Acc:HGNC:1784] | 6317 | 0.811 | 0.3217 | Yes |
| NR4A2 | nuclear receptor subfamily 4 group A member 2 [Source:HGNC Symbol;Acc:HGNC:7981] | 6366 | 0.8 | 0.3263 | Yes |
| CD83 | CD83 molecule [Source:HGNC Symbol;Acc:HGNC:1703] | 6514 | 0.783 | 0.3258 | Yes |
| GADD45B | growth arrest and DNA damage inducible beta [Source:HGNC Symbol;Acc:HGNC:4096] | 6517 | 0.783 | 0.3325 | Yes |
| NFIL3 | nuclear factor, interleukin 3 regulated [Source:HGNC Symbol;Acc:HGNC:7787] | 6542 | 0.778 | 0.3381 | Yes |
| BCL3 | BCL3 transcription coactivator [Source:HGNC Symbol;Acc:HGNC:998] | 6558 | 0.776 | 0.3442 | Yes |
| PLAU | plasminogen activator, urokinase [Source:HGNC Symbol;Acc:HGNC:9052] | 6669 | 0.763 | 0.3453 | Yes |
| EFNA1 | ephrin A1 [Source:HGNC Symbol;Acc:HGNC:3221] | 6680 | 0.761 | 0.3515 | Yes |
| LITAF | lipopolysaccharide induced TNF factor [Source:HGNC Symbol;Acc:HGNC:16841] | 6785 | 0.749 | 0.3528 | Yes |
| GADD45A | growth arrest and DNA damage inducible alpha [Source:HGNC Symbol;Acc:HGNC:4095] | 6822 | 0.746 | 0.3575 | Yes |
| KLF6 | KLF transcription factor 6 [Source:HGNC Symbol;Acc:HGNC:2235] | 6830 | 0.744 | 0.3637 | Yes |
| DUSP2 | dual specificity phosphatase 2 [Source:HGNC Symbol;Acc:HGNC:3068] | 7256 | 0.694 | 0.3483 | No |
| CFLAR | CASP8 and FADD like apoptosis regulator [Source:HGNC Symbol;Acc:HGNC:1876] | 7559 | 0.684 | 0.3391 | No |
| KLF2 | KLF transcription factor 2 [Source:HGNC Symbol;Acc:HGNC:6347] | 7636 | 0.684 | 0.3413 | No |
| HES1 | hes family bHLH transcription factor 1 [Source:HGNC Symbol;Acc:HGNC:5192] | 7657 | 0.684 | 0.3463 | No |
| STAT5A | signal transducer and activator of transcription 5A [Source:HGNC Symbol;Acc:HGNC:11366] | 7679 | 0.684 | 0.3512 | No |
| RCAN1 | regulator of calcineurin 1 [Source:HGNC Symbol;Acc:HGNC:3040] | 7843 | 0.678 | 0.3489 | No |
| SPHK1 | sphingosine kinase 1 [Source:HGNC Symbol;Acc:HGNC:11240] | 8352 | 0.594 | 0.3286 | No |
| TNFAIP8 | TNF alpha induced protein 8 [Source:HGNC Symbol;Acc:HGNC:17260] | 8406 | 0.583 | 0.331 | No |
| CCL20 | C-C motif chemokine ligand 20 [Source:HGNC Symbol;Acc:HGNC:10619] | 8626 | 0.564 | 0.3249 | No |
| SGK1 | serum/glucocorticoid regulated kinase 1 [Source:HGNC Symbol;Acc:HGNC:10810] | 8641 | 0.564 | 0.3292 | No |
| TNFSF9 | TNF superfamily member 9 [Source:HGNC Symbol;Acc:HGNC:11939] | 8656 | 0.564 | 0.3334 | No |
| TNFAIP3 | TNF alpha induced protein 3 [Source:HGNC Symbol;Acc:HGNC:11896] | 8662 | 0.564 | 0.3381 | No |
| EDN1 | endothelin 1 [Source:HGNC Symbol;Acc:HGNC:3176] | 8665 | 0.564 | 0.3429 | No |
| IL23A | interleukin 23 subunit alpha [Source:HGNC Symbol;Acc:HGNC:15488] | 8751 | 0.553 | 0.3435 | No |
| IRF1 | interferon regulatory factor 1 [Source:HGNC Symbol;Acc:HGNC:6116] | 8884 | 0.537 | 0.3416 | No |
| SERPINB8 | serpin family B member 8 [Source:HGNC Symbol;Acc:HGNC:8952] | 8894 | 0.535 | 0.3458 | No |
| NFKB1 | nuclear factor kappa B subunit 1 [Source:HGNC Symbol;Acc:HGNC:7794] | 8958 | 0.526 | 0.3472 | No |
| TNIP1 | TNFAIP3 interacting protein 1 [Source:HGNC Symbol;Acc:HGNC:16903] | 8985 | 0.523 | 0.3505 | No |
| PNRC1 | proline rich nuclear receptor coactivator 1 [Source:HGNC Symbol;Acc:HGNC:17278] | 9030 | 0.518 | 0.3528 | No |
| TGIF1 | TGFB induced factor homeobox 1 [Source:HGNC Symbol;Acc:HGNC:11776] | 9133 | 0.5 | 0.3521 | No |
| SERPINE1 | serpin family E member 1 [Source:HGNC Symbol;Acc:HGNC:8583] | 9273 | 0.489 | 0.3493 | No |
| SDC4 | syndecan 4 [Source:HGNC Symbol;Acc:HGNC:10661] | 9578 | 0.48 | 0.3383 | No |
| CCRL2 | C-C motif chemokine receptor like 2 [Source:HGNC Symbol;Acc:HGNC:1612] | 9595 | 0.48 | 0.3417 | No |
| CEBPD | CCAAT enhancer binding protein delta [Source:HGNC Symbol;Acc:HGNC:1835] | 9640 | 0.477 | 0.3436 | No |
| ZC3H12A | zinc finger CCCH-type containing 12A [Source:HGNC Symbol;Acc:HGNC:26259] | 9779 | 0.472 | 0.3408 | No |
| KYNU | kynureninase [Source:HGNC Symbol;Acc:HGNC:6469] | 9987 | 0.462 | 0.3344 | No |
| MAFF | MAF bZIP transcription factor F [Source:HGNC Symbol;Acc:HGNC:6780] | 10027 | 0.457 | 0.3365 | No |
| CSF2 | colony stimulating factor 2 [Source:HGNC Symbol;Acc:HGNC:2434] | 10031 | 0.457 | 0.3403 | No |
| TANK | TRAF family member associated NFKB activator [Source:HGNC Symbol;Acc:HGNC:11562] | 10231 | 0.449 | 0.3342 | No |
| KDM6B | lysine demethylase 6B [Source:HGNC Symbol;Acc:HGNC:29012] | 10442 | 0.449 | 0.3276 | No |
| ZBTB10 | zinc finger and BTB domain containing 10 [Source:HGNC Symbol;Acc:HGNC:30953] | 10825 | 0.444 | 0.3123 | No |
| BTG3 | BTG anti-proliferation factor 3 [Source:HGNC Symbol;Acc:HGNC:1132] | 10927 | 0.436 | 0.311 | No |
| IL6 | interleukin 6 [Source:HGNC Symbol;Acc:HGNC:6018] | 11025 | 0.433 | 0.3099 | No |
| DUSP4 | dual specificity phosphatase 4 [Source:HGNC Symbol;Acc:HGNC:3070] | 11040 | 0.433 | 0.313 | No |
| SPSB1 | splA/ryanodine receptor domain and SOCS box containing 1 [Source:HGNC Symbol;Acc:HGNC:30628] | 11082 | 0.433 | 0.3147 | No |
| TRIP10 | thyroid hormone receptor interactor 10 [Source:HGNC Symbol;Acc:HGNC:12304] | 11169 | 0.433 | 0.3142 | No |
| SMAD3 | SMAD family member 3 [Source:HGNC Symbol;Acc:HGNC:6769] | 11223 | 0.432 | 0.3153 | No |
| ICOSLG | inducible T cell costimulator ligand [Source:HGNC Symbol;Acc:HGNC:17087] | 11352 | 0.425 | 0.3126 | No |
| IL15RA | interleukin 15 receptor subunit alpha [Source:HGNC Symbol;Acc:HGNC:5978] | 11585 | 0.423 | 0.3046 | No |
| EGR2 | early growth response 2 [Source:HGNC Symbol;Acc:HGNC:3239] | 11784 | 0.409 | 0.2982 | No |
| IER2 | immediate early response 2 [Source:HGNC Symbol;Acc:HGNC:28871] | 11875 | 0.395 | 0.2972 | No |
| TNF | tumor necrosis factor [Source:HGNC Symbol;Acc:HGNC:11892] | 11928 | 0.389 | 0.298 | No |
| PDE4B | phosphodiesterase 4B [Source:HGNC Symbol;Acc:HGNC:8781] | 11942 | 0.388 | 0.3007 | No |
| TRAF1 | TNF receptor associated factor 1 [Source:HGNC Symbol;Acc:HGNC:12031] | 12059 | 0.38 | 0.2982 | No |
| KLF4 | KLF transcription factor 4 [Source:HGNC Symbol;Acc:HGNC:6348] | 12119 | 0.376 | 0.2985 | No |
| TNFAIP6 | TNF alpha induced protein 6 [Source:HGNC Symbol;Acc:HGNC:11898] | 12426 | 0.356 | 0.2862 | No |
| PTPRE | protein tyrosine phosphatase receptor type E [Source:HGNC Symbol;Acc:HGNC:9669] | 12550 | 0.35 | 0.2831 | No |
| TUBB2A | tubulin beta 2A class IIa [Source:HGNC Symbol;Acc:HGNC:12412] | 12605 | 0.346 | 0.2834 | No |
| GFPT2 | glutamine-fructose-6-phosphate transaminase 2 [Source:HGNC Symbol;Acc:HGNC:4242] | 12744 | 0.332 | 0.2794 | No |
| NAMPT | nicotinamide phosphoribosyltransferase [Source:HGNC Symbol;Acc:HGNC:30092] | 12795 | 0.327 | 0.2797 | No |
| PTX3 | pentraxin 3 [Source:HGNC Symbol;Acc:HGNC:9692] | 12834 | 0.322 | 0.2806 | No |
| IL6ST | interleukin 6 cytokine family signal transducer [Source:HGNC Symbol;Acc:HGNC:6021] | 12846 | 0.32 | 0.2829 | No |
| RELB | RELB proto-oncogene, NF-kB subunit [Source:HGNC Symbol;Acc:HGNC:9956] | 13054 | 0.302 | 0.2751 | No |
| BCL6 | BCL6 transcription repressor [Source:HGNC Symbol;Acc:HGNC:1001] | 13098 | 0.3 | 0.2756 | No |
| NR4A1 | nuclear receptor subfamily 4 group A member 1 [Source:HGNC Symbol;Acc:HGNC:7980] | 13409 | 0.288 | 0.2625 | No |
| PLEK | pleckstrin [Source:HGNC Symbol;Acc:HGNC:9070] | 13528 | 0.278 | 0.259 | No |
| NFKBIA | NFKB inhibitor alpha [Source:HGNC Symbol;Acc:HGNC:7797] | 13622 | 0.278 | 0.2567 | No |
| CCNL1 | cyclin L1 [Source:HGNC Symbol;Acc:HGNC:20569] | 13804 | 0.272 | 0.25 | No |
| ABCA1 | ATP binding cassette subfamily A member 1 [Source:HGNC Symbol;Acc:HGNC:29] | 13835 | 0.272 | 0.2509 | No |
| PDLIM5 | PDZ and LIM domain 5 [Source:HGNC Symbol;Acc:HGNC:17468] | 13871 | 0.27 | 0.2515 | No |
| PTGS2 | prostaglandin-endoperoxide synthase 2 [Source:HGNC Symbol;Acc:HGNC:9605] | 13943 | 0.269 | 0.2503 | No |
| GEM | GTP binding protein overexpressed in skeletal muscle [Source:HGNC Symbol;Acc:HGNC:4234] | 14044 | 0.268 | 0.2476 | No |
| IL1A | interleukin 1 alpha [Source:HGNC Symbol;Acc:HGNC:5991] | 14049 | 0.268 | 0.2497 | No |
| NR4A3 | nuclear receptor subfamily 4 group A member 3 [Source:HGNC Symbol;Acc:HGNC:7982] | 14110 | 0.267 | 0.249 | No |
| CD44 | CD44 molecule (Indian blood group) [Source:HGNC Symbol;Acc:HGNC:1681] | 14307 | 0.254 | 0.2414 | No |
| SERPINB2 | serpin family B member 2 [Source:HGNC Symbol;Acc:HGNC:8584] | 14842 | 0.239 | 0.2166 | No |
| TNIP2 | TNFAIP3 interacting protein 2 [Source:HGNC Symbol;Acc:HGNC:19118] | 14913 | 0.239 | 0.2152 | No |
| CD80 | CD80 molecule [Source:HGNC Symbol;Acc:HGNC:1700] | 15010 | 0.239 | 0.2125 | No |
| NFKBIE | NFKB inhibitor epsilon [Source:HGNC Symbol;Acc:HGNC:7799] | 15022 | 0.239 | 0.214 | No |
| IRS2 | insulin receptor substrate 2 [Source:HGNC Symbol;Acc:HGNC:6126] | 15073 | 0.235 | 0.2135 | No |
| TAP1 | transporter 1, ATP binding cassette subfamily B member [Source:HGNC Symbol;Acc:HGNC:43] | 15212 | 0.228 | 0.2086 | No |
| RIGI | RNA sensor RIG-I [Source:HGNC Symbol;Acc:HGNC:19102] | 15236 | 0.227 | 0.2094 | No |
| PLAUR | plasminogen activator, urokinase receptor [Source:HGNC Symbol;Acc:HGNC:9053] | 15300 | 0.225 | 0.2082 | No |
| PMEPA1 | prostate transmembrane protein, androgen induced 1 [Source:HGNC Symbol;Acc:HGNC:14107] | 15627 | 0.21 | 0.1936 | No |
| ETS2 | ETS proto-oncogene 2, transcription factor [Source:HGNC Symbol;Acc:HGNC:3489] | 15650 | 0.207 | 0.1944 | No |
| BMP2 | bone morphogenetic protein 2 [Source:HGNC Symbol;Acc:HGNC:1069] | 15831 | 0.189 | 0.1869 | No |
| SQSTM1 | sequestosome 1 [Source:HGNC Symbol;Acc:HGNC:11280] | 16038 | 0.183 | 0.1782 | No |
| IL12B | interleukin 12B [Source:HGNC Symbol;Acc:HGNC:5970] | 16098 | 0.183 | 0.1768 | No |
| PLK2 | polo like kinase 2 [Source:HGNC Symbol;Acc:HGNC:19699] | 16445 | 0.168 | 0.1609 | No |
| FOSL1 | FOS like 1, AP-1 transcription factor subunit [Source:HGNC Symbol;Acc:HGNC:13718] | 16529 | 0.166 | 0.1581 | No |
| IER5 | immediate early response 5 [Source:HGNC Symbol;Acc:HGNC:5393] | 16587 | 0.163 | 0.1567 | No |
| NFAT5 | nuclear factor of activated T cells 5 [Source:HGNC Symbol;Acc:HGNC:7774] | 16588 | 0.163 | 0.1581 | No |
| IFIH1 | interferon induced with helicase C domain 1 [Source:HGNC Symbol;Acc:HGNC:18873] | 16648 | 0.157 | 0.1565 | No |
| ICAM1 | intercellular adhesion molecule 1 [Source:HGNC Symbol;Acc:HGNC:5344] | 16680 | 0.154 | 0.1563 | No |
| CCL4 | C-C motif chemokine ligand 4 [Source:HGNC Symbol;Acc:HGNC:10630] | 16819 | 0.151 | 0.1507 | No |
| CCND1 | cyclin D1 [Source:HGNC Symbol;Acc:HGNC:1582] | 17457 | 0.13 | 0.1198 | No |
| SLC2A6 | solute carrier family 2 member 6 [Source:HGNC Symbol;Acc:HGNC:11011] | 17520 | 0.127 | 0.1178 | No |
| VEGFA | vascular endothelial growth factor A [Source:HGNC Symbol;Acc:HGNC:12680] | 17877 | 0.109 | 0.1008 | No |
| LAMB3 | laminin subunit beta 3 [Source:HGNC Symbol;Acc:HGNC:6490] | 17943 | 0.106 | 0.0985 | No |
| PFKFB3 | 6-phosphofructo-2-kinase/fructose-2,6-biphosphatase 3 [Source:HGNC Symbol;Acc:HGNC:8874] | 18060 | 0.1 | 0.0935 | No |
| IFIT2 | interferon induced protein with tetratricopeptide repeats 2 [Source:HGNC Symbol;Acc:HGNC:5409] | 18069 | 0.1 | 0.094 | No |
| MXD1 | MAX dimerization protein 1 [Source:HGNC Symbol;Acc:HGNC:6761] | 18285 | 0.087 | 0.0839 | No |
| PPP1R15A | protein phosphatase 1 regulatory subunit 15A [Source:HGNC Symbol;Acc:HGNC:14375] | 18454 | 0.083 | 0.0762 | No |
| TNC | tenascin C [Source:HGNC Symbol;Acc:HGNC:5318] | 18549 | 0.08 | 0.0722 | No |
| SLC2A3 | solute carrier family 2 member 3 [Source:HGNC Symbol;Acc:HGNC:11007] | 18632 | 0.078 | 0.0687 | No |
| B4GALT1 | beta-1,4-galactosyltransferase 1 [Source:HGNC Symbol;Acc:HGNC:924] | 18727 | 0.072 | 0.0647 | No |
| LDLR | low density lipoprotein receptor [Source:HGNC Symbol;Acc:HGNC:6547] | 18769 | 0.069 | 0.0632 | No |
| IL7R | interleukin 7 receptor [Source:HGNC Symbol;Acc:HGNC:6024] | 18793 | 0.068 | 0.0626 | No |
| SNN | stannin [Source:HGNC Symbol;Acc:HGNC:11149] | 18924 | 0.059 | 0.0566 | No |
| JAG1 | jagged canonical Notch ligand 1 [Source:HGNC Symbol;Acc:HGNC:6188] | 19443 | 0.03 | 0.0308 | No |
| SOD2 | superoxide dismutase 2 [Source:HGNC Symbol;Acc:HGNC:11180] | 19839 | 0.005 | 0.011 | No |
